# Supplementary material for: Hyaluronic Acid Oligosaccharide Derivatives Alleviate Lipopolysaccharide-Induced Inflammation in ATDC5 Cells by Multiple Mechanisms
Source: Molecules. 2022 Aug 31;27(17):5619. doi: 10.3390/molecules27175619 (PMC9457626; doi:10.3390/molecules27175619)
Supplement: Supplementary file 1 [file molecules-27-05619-s001.zip › Supplementary file-final version-0904.pdf]

Supplementary Information

Hyaluronic Acid Oligosaccharide Derivatives Alleviate Lipopolysaccharide-induced Inflammation in ATDC5 Cells by Multiple Mechanisms

Hesuyuan Huang[1,2†], Xuyang Ding[3†], Dan Xing[1,2], Jianjing Lin[1,2], Zhongtang Li[3*], Jianhao Lin[1,2*]

[1]Arthritis Clinic & Research Center, Peking, University People's Hospital, Peking, University, Beijing, China

[2]Arthritis Institute, Peking University, Beijing, China

[3]State Key Laboratory of Natural and Biomimetic Drugs, School of Pharmaceutical Sciences, Peking University, Beijing, 100191, China

[*]Correspondence: lizhongtang@bjmu.edu.cn (Z.L.); linjianhao@pkuph.edu.cn (J.L.)

[†]These authors contributed equally to this work.

Table of contents

Materials and Methods S1 The detailed synthesis procedures of glycosides of HA oligosaccharides and its derivatives

Figure S1 ^{1}H NMR, ^{13}C NMR， HRMS and HPLC spectra of the compounds **2-15**.
Table S1 HPLC of compounds **6, 7, 10, 11, 14, 15**.
Figure S2 o-HA derivatives had no pro-inflammatory effect on THP-1 cells.
Figure S3 Different HA derivatives down-regulated IL-1β and IL-6 protein levels in LPS-induced inflammatory injury in ATDC5 cells.
Figure S4 Different HA derivatives alleviated the inflammatory injury of THP-1 cells induced by LPS.
Figure S5 The ratio of LC3-II/LC3-I was determined by Western blot analysis after using autophagy inhibitor.

Materials and Methods S1 The detailed synthesis procedures of glycosides of HA oligosaccharides

Method A: General Procedure for Deprotection.

A solution of acetylated derivatives (~0.30 mmol for the dimer, ∼0.17 mmol for the tetramer, ∼0.11 mmol for the hexamer) in a mixed solvent of THF/H_2O = 3/1 (6.0 mL) with a stir bar was cooled to -5 °C. Then a freshly prepared mixture of 1 M LiOH (aq.)/H_2O_2 (35% wt) = 2:1 (*v*/*v*, 2.25mL) was added. The colorless solution was stirred at -5 °C for 1 h, then warmed to room temperature (rt), and stirred for another 16 h. Without monitoring by TLC, the mixture was cooled to 0 °C (ice/water bath), followed by the addition of MeOH (2.1 mL) and 4 M NaOH (aq., 4 mL). The mixture was slowly warmed to room temperature and vigorously stirred for 12h. The reaction solution was neutralized by Amberlite IR-120 [H^+] resin, filtered, concentrated, and then purified by the Sephadex LH-20 eluent with H_2O. The fractions containing the desired product (TLC monitored) were combined and then lyophilized to afford the corresponding oligosaccharides.

O-(Methyl 2, 3, 4-tri-O-acetyl-β-D-glucopyranosyluronate)-(1→3)-1, 4, 6-tri-O-acetyl-2-deoxy-2-acetamido-D- glucopyranose (2)

A solution of sodium hyaluronate (10g, Mw.~500kDa) in water (285ml) was stired at 80 ºC until swelled completely, and then hydrochloric acid (11.74 M, 15.2 mL) was added dropwise to a volume of 300mL (final concentration as 0.5M). The mixture was stirred at 80 ºC for 2 days, monitored by TLC until completely hydrolysis, followed by cooling to room temperature. The mixture was adjusted to pH = 7.0 with NaOH solid under vigorous stirring. The solution was concentrated under reduced pressure to 20 mL, slowly droped the solution into 1000 mL EtOH while vigorously stirring to obtain a light brown precipitation, filtered, and dried the precipitation to obtain a yellow powder. The powder was treated with HCl/MeOH (0.02 M, 200 mL) for 4 days at 4ºC, monitored by TLC until complete reaction and then the solution was neutralized by

trimethylamine (Et_3N), concentrated under reduced pressure to obtain yellow powder. The yellow powder was dissolved in 50 mL dichloromethane (DCM), 298mg dimethylaminopyridine (DMAP), 15mL Et_3N were added, 50mL acetic anhydride (Ac_2O) was added dropwise under ice bath, and it was naturally warmed to room temperature. After the reaction was completed, water was added to quench under ice bath, and the organic phase was separated, washed with saturated aqueous $NaHCO_3$, and saturated brine, dried over anhydrous Na_2SO_4, and concentrated. Through silica gel chromatography separation (DCM/MeOH=60/1, containing 0.1% Et_3N) , 5.20g of light yellow disaccharide **2** was obtained. (α/β=2.5/1, isomers was confirmed by ^{1}H NMR, three-step overall yield: 31%). R_f=0.40(DCM/MeOH=20/1).

O-(Methyl 2, 3, 4-tri-O-acetyl-β-D-glucopyranosyluronate)-(1→3)-2-methyl-(4, 6di-O-acetyl-1, 2-dideoxy-α-D glucopyranose)[2, 1-d] 2-oxazoline(3)

A solution of **2** (5.20 g, 7.84 mmol, 1.0 eq) in 180 mL anhydrous DCM was added dropwise trimethylsilyl trifluoromethylsulfonate (TMSOTf, 2.84 mL, 15.68 mmol, 2.0 eq) at 0 °C under argon atmosphere. The reaction mixture was stirred at rt until the starting material was totally consumed, quenched with Et_3N at 0 °C, and evaporated. The residue was purified by silica gel chromatography (petroleum ether/ethyl acetate=1/1.7~1/2.0, containing 0.1% Et_3N) to give pure **3** (4.91 g, 82%) as white amorphous. R_f = 0.40 (DCM/MeOH= 30/1). ^{1}H NMR (400MHz, $CDCl_3$, TMS) δ6.00 (1H, d, *J*=6.93Hz), δ5.29 (1H, t, *J*=9.42Hz), δ5.23 (2H, t, *J*=9.37Hz), δ5.01(1H, t, *J*=8.26Hz), δ5.01(1H, t, *J*=8.26Hz), δ4.94(1H, d, *J*=8.01Hz), δ4.20-4.05(5H, m), δ3.76(3H, s), δ3.63(1H, m, *J*=4.33Hz), 2.08-2.02 (18H, m) ppm. ^{13}C NMR (100 MHz, $CDCl_3$, TMS) δ170.78, 170.09, 169.93, 169.37, 169.18, 166.92, 100.78, 99.84, 78.30, 77.22, 72.34, 72.21, 71.20, 69.16, 67.77, 67.54, 63.57, 52.81, 20.93, 20.73, 20.61, 20.59, 20.49, 13.99 ppm.

Azido O-(Methyl 2, 3, 4-tri-O-acetyl-β-D-glucopyranosyluronate)-(1→3)-2-methyl-(4, 6di-O-acetyl-1, 2-dideoxy-α-D glucopyranose) (4)

A solution of **3** (2 g, 3.31 mmol) in anhydrous $CHCl_3$ (20 mL) was added TMS-N_3(3.48mL, 26.48 mmol, 8 eq), anhydrous Cupric (II) chloride (625 mg, 3.64 mmol, 1.1 eq) under argon atmosphere. The mixture was refluxed overnight, quenched by saturated aqueous $NaHCO_3$ and the solids were filtered off through a Celite pad. The

filtrate was extracted with DCM and the organic phase was dried over Na_2SO_4, concentrated, and purified by silica gel chromatography (DCM/MeOH=50/1, containing 0.1% Et_3N) to give pure **4** (1.76g, 82%) as white solid. R_f= 0.45 (DCM/ MeOH= 20/1). ^{1}H NMR (400MHz, $CDCl_3$, TMS) δ6.06 (1H, d, *J*=7.73Hz), δ5.31-5.10 (3H, m), δ4.98(1H, t, *J*=9.55Hz), δ4.85(1H, t, *J*=8.31Hz), δ4.71(1H, d, *J*=7.47Hz), δ4.48(1H, t, *J*=9.96Hz), δ4.25(1H, dd, J_1=4.98Hz, J_2=12.46Hz), δ4.13(1H, dd, *J*=2.24Hz, *J*=12.46Hz), δ4.02(1H, d, *J*=9.93Hz), δ3.81-3.72(4H, m), δ3.27(1H, q, *J*=8.48Hz), δ2.11-2.00(18H, m) ppm. ^{13}C NMR (100 MHz, $CDCl_3$, TMS) δ171.17, 170.77, 170.16, 169.65, 169.37, 169.08, 167.08, 99.63, 87.10, 77.35, 77.03, 76.71, 73.92, 72.19, 72.10, 71.82, 69.33, 67.85, 62.10, 57.25, 52.83, 23.56, 20.76, 20.71, 20.61, 20.56, 20.44 ppm.

Methyl O-(Methyl 2, 3, 4-tri-O-acetyl-β-D-glucopyranosyluronate)-(1→3)-2-methyl-(4, 6di-O-acetyl-1, 2-dideoxy-α-D glucopyranose) (5)

A solution of **3** (2 g, 3.31 mmol) in anhydrous $CHCl_3$ (20 mL) was added anhydrous MeOH (1.07mL,26.48 mmol, 8 eq), anhydrous Cupric (II) chloride (625 mg, 3.64 mmol, 1.1 eq) under argon atmosphere. The mixture was refluxed overnight, quenched by saturated aqueous $NaHCO_3$ and the solids were filtered off through a Celite pad. The filtrate was extracted with DCM and the organic phase was dried over Na_2SO_4, concentrated, and purified by silica gel chromatography (DCM/MeOH=50:1, containing 0.1%Et_3N) to give pure **5** (1.79g, 85%) as white solid. R_f = 0.35 (DCM/ MeOH=20/1). ^{1}H NMR(400MHz, $CDCl_3$, TMS) δ6.07 (1H, d,*J*=7.27Hz), δ5.20 (1H, t, *J*=9.24Hz), δ5.12 (1H, t, *J*=9.85Hz), δ4.94 (1H, t, *J*=9.44Hz), δ4.80-4.87 (2H, m), δ4.68 (1H, d, *J*=7.67Hz), δ4.48 (1H, t, *J*=9.53Hz), δ4.23 (1H, dd, J_1=5.03Hz, J_2 = 11.64Hz), δ4.08 (1H, dd, J_1= 2.51Hz, J_2=12.04Hz), δ3.99 (1H, d, *J*= 9.79Hz), δ3.70 (3H, s) , δ3.66 (1H, m, *J*= 2.25Hz), δ3.46 (3H, s), δ3.22 (1H, m, *J*= 10.05Hz), δ2.09- 1.95 (18H, m) ppm. ^{13}C NMR (100 MHz, $CDCl_3$, TMS) 171.17, 170.81, 170.13, 169.74, 169.37, 169.09, 167.09, 100.14, 99.76, 72.22, 71.74, 69.43, 68.43, 62.39, 57.66, 56.96, 52.73, 23.64, 20.77, 20.67, 20.64, 20.55, 20.43 ppm.

Azido O-(β-D-glucopyranosyluronate)-(1→3)-2-acetamido-2-deoxy-β-D-

glucopyranoside (6)

Compound **6** was obtained from **4** (200 mg) according to Method A. (yield 104.3 mg, 85%). R_f= 0.60 (n-BuOH/H_2O/EtOH/AcOH = 1/1/1/0.05). ^1H NMR (400MHz, D_2O, TMS) δ4.40(1H, d, *J*=7.72Hz), δ3.85-3.62(5H, m), δ3.52-3.37(4H, m), δ3.31-3.21(2H, m), δ1.95(1H, s) ppm. ^{13}C NMR (100 MHz, D_2O, TMS) δ175.48, 174.83, 102.86, 88.49, 82.37, 77.49, 75.79, 75.32, 72.67, 71.68, 68.32, 60.54, 53.93, 48.88, 22.17 ppm. HRMS (ESI, negetive mode) calculated for $C_{14}H_{21}N_4O_{11}^-$ $[M]^-$ m/z 421.1212, found 421.1208.

Methyl O-(β-D-glucopyranosyluronate)-(1→3)-2-acetamido-2-deoxy-β-D – glucopyranoside (7)

Compound **7** was obtained from **5** (200 mg) according to Method A. (yield 111mg, 86%). R_f= 0.55 (n-BuOH/H_2O/EtOH/AcOH = 1/1/1/0.05). ^1H NMR (400MHz, D_2O, TMS) δ4.41(2H, d, *J*=8.01Hz), δ3.88(1H, dd, *J* = 2.10Hz, *J*=12.49Hz), δ3.81-3.64(4H, m), δ3.51- 3.38(7H, m), δ3.28(1H, m), δ1.96 (3H, s) ppm. ^{13}C NMR (100 MHz, D_2O, TMS) δ175.43, 174.86, 103.02, 101.83, 83.11, 75.51, 72.86, 71.78, 68.95, 60.92, 57.21, 54.44, 22.37 ppm. HRMS (ESI, negetive mode) calculated for $C_{15}H_{24}N_1O_{12}^-$ $[M]^-$ m/z 410.1304, found 410.1303.

HA Enzymatic degradation mixture: Tetrasccharide and Hexasaccharide

Sodium hyaluronate (MW. ~500 kDa) 10 g was suspended into sodium acetate buffer (1000 mL, 0.15 M NaCl, 0.10 M NaOAc, adjusted to pH = 5.0 by AcOH). The mixture was stirred at 37 ºC until the solid swelled completely to give a colorless gel. The gel was warmed to 37 ºC in a water bath. Hyaluronidase from bovine testes 200mg was added in one portion and the mixture was kept at 37 ºC for 7 days. After the reaction was completed (TLC monitored), the solution was boiled to 100℃, then slowly cooled at room temperature to afford a turbid yellow solution. The solution was filtered through a small pad of Celite® (4 cm × 5 cm) under reduced pressure. The solution was concentrated under reduced pressure to 20 mL, slowly droped the solution into 1000 mL EtOH to obtain a white precipitation, filtered , and dried the precipitation to obtain a dry white powder crude product of **8** (6.6 g).

Azido O-(methyl 2,3,4-tri-O-acetyl-β-D-glucopyranosyluronate)-(1→3)-(4,6-di-O-acetyl-2-acetamido-2-deoxy-β-D-glucopyranoside)-(1→4)-(methyl 2,3-di-O-acetyl-β-D-glucopyranosyluronate)-(1→3)-4,6-di-O-acetyl-2-acetamido-2-deoxy-D-glucopyranoside (8) and Azido O-(methyl 2,3,4-tri-O-acetyl-β-D-glucopyranosyluronate)-(1→3)-(4,6-di-O-acetyl-2-acetamido-2-deoxy-β-D-glucopyranoside)-(1→4)-(methyl 2,3-di-O-acetyl-β-D-glucopyranosyluronate)-(1→3)-(4,6-di-O-acetyl-2-acetamido-2-deoxy-D-glucopyranoside)-(1→4)-(methyl 2,3-di-O-acetyl-β-D-glucopyranosyluronate)-(1→3)-4,6-di-O-acetyl-2-acetamido-2-deoxy-D-glucopyranoside (9)

3.3g HA enzymolysis mixture (4.25mmol, calculated according to molecular weight of tetrasaccharide), 10.6 mL N-methylmorpholine (212 mmol, 50eq), and 13.8g NaN_3 (212mmol, 50eq) were dissolved in 75mL water, and 7.2 g DMC was added under ice bath, stirred for 15 minutes, removed the ice bath, and naturally warmed to room temperature to continue stir for 36 hours, monitored by TLC until completely reaction. The solution was concentrated under reduced pressure, purified by the Sephadex LH-20 eluent with H_2O. The eluant was combined and concentrated under reduced pressure, treated with HCl/MeOH (0.06 M, 330 mL) for 4 d at 4ºC, monitored by TLC until complete reaction and then the solution was neutralized by Et_3N, concentrated under reduced pressure to obtain pink powder. The pink powder was dissolved in 30mL DCM, 179mg DMAP, 9mL Et_3N were added, 30mL Ac_2O was added dropwise under ice bath, and it was naturally warmed to rt. After the reaction was completed, water was added to quench under ice bath, and the organic phase was separated, washed with saturated aqueous $NaHCO_3$, and saturated brine, dried over anhydrous Na_2SO_4, and concentrated. Through silica gel chromatography separation (DCM/ EtOAc/ MeOH=1/1/0.04 to1/1/0.06, containing 0.1%Et_3N), 1.6g **8** was obtained as white solid and 1.3g **9** was obtained as white solid. **8**: R_f= 0.35 (DCM/EA/MeOH=1/1/0.2) and **9**: R_f= 0.30(DCM/EA/MeOH=1/1/0.2)

Compound 8:^{1}H NMR(600MHz,$CDCl_3$, TMS) δ5.92(2H, d, *J*=6.71Hz), δ5.20(1H, t, *J*= 9.05 Hz), δ5.15-5.04(3H, m), δ4.90(2H, m), δ4.86- 4.77(3H, m), δ4.65(1H, d,

J= 7.38 Hz), δ4.61(1H, d, J=8.05Hz), δ4.51(1H, t, J= 9.39Hz), δ4.37(2H, t, J= 10.06 Hz), δ4.32 (1H, dd, J_1=4.01Hz, J_2=12.41Hz) ,δ4.23(1H, dd, J_1=4.88Hz, J_2=12.73Hz), δ3.99-3.97(1H, d, J=2.56Hz), δ3.97-3.95(1H, d, J=3.76Hz), δ3.82(3H, s), δ3.77-3.73 (1H, m), δ3.71(3H, s), δ3.66-3.62(1H, m), δ3.30-3.20(1H, m), δ2.09-1.97 (33 H, m) ppm. ^{13}C NMR (150 MHz, $CDCl_3$, TMS) δ171.20, 171.18, 170.78, 170.68, 170.19, 170.04, 169.73, 169.34, 169.12, 169.02, 167.77, 166.96, 100.65, 100.02, 98.32, 87.11, 77.57, 77.24, 77.03, 76.82, 76.58, 75.61, 74.63, 73.93, 72.32, 72.18, 71.92, 71.87, 71.82, 69.41, 68.32, 68.11, 62.08, 61.98, 60.42, 58.15, 57.25, 53.02, 52.74, 23.63, 23.57, 21.07, 20.77, 20.74, 20.59, 20.53, 20.52, 20.46, 14.20 ppm.

Compound 9:^{1}H NMR(600MHz,$CDCl_3$, TMS) δ6.10(1H, d, J=5.81Hz), δ6.01(1H, d, J= 5.36Hz) , δ5.81(1H, d, J=6.26Hz), δ5.20(1H, t, J=9.36Hz), δ6.13(1H, d, J= 9.39Hz), δ5.10-5.01(3H, m), δ4.92(1H, t, J=9.39Hz), δ4.87(1H, t, J=8.94Hz), δ4.86-4.76(6H, m), δ4.68(1H, d, J=8.05Hz), δ4.61(1H, d, J=7.60Hz), δ4.55(1H, d, J=7.98Hz), δ4.50(1H, t, J=9.75Hz), δ4.44(1H, t, J=9.31Hz), δ4.37- 4.28(3H, m), δ4.23(1H, dd, J_1=4.65Hz, J_2=12.85Hz), δ4.14(1H, d, J=11.30Hz), δ4.09-3.94 (6H, m), δ3.92(1H, d, J=3.91Hz), δ3.82(3H, s), δ3.81(3H, s), δ3.76(1H, m), δ3.71(3H, s), δ3.63(1H, m), δ3.33(1H, m), δ2.11-1.98(48H, m)ppm. ^{13}C NMR (150 MHz, $CDCl_3$, TMS) δ171.27, 171.24, 171.14, 170.83, 170.71, 170.68, 170.19, 170.16, 169.97, 169.75, 169.36, 169.24, 169.03, 167.81, 167.76, 166.98, 100.90, 100.63, 100.02, 98.46, 98.37, 87.28, 77.74, 76.61, 75.70, 75.62, 74.68, 74.51, 73.94, 72.31, 72.23, 72.19, 71.86, 71.77, 71.62, 69.42, 68.42, 68.35, 68.13, 62.12, 62.02, 61.97, 58.11, 56.98, 53.01, 52.76, 30.98, 23.63, 23.56, 20.79, 20.77, 20.61, 20.55, 20.52, 20.47 ppm.

Azido O-(β-D-glucopyranosyluronate)-(1→3)-(2-acetamido-2-deoxy-β-D –glucopyranoside)-(1→4)- (β-D-glucopyranosyluronate)-(1→3)-2-acetamido-2-deoxy-β-D –glucopyranoside (10)

Compound **10** was obtained from **8** (200 mg) according to Method A. (yield 116.6mg, 85%). R_f= 0.45 (n-BuOH/H_2O/EtOH/AcOH = 1/1/1/0.05)^{1}H NMR (600MHz, D_2O, TMS) δ4.55(1H, d, J=8.56Hz), δ4.47 (2H,dd, J=7.77Hz, J= 12.25Hz), δ3.90(2H, d, J=11.75Hz), δ3.84(2H, t, J=9.71Hz), δ3.80-3.67(7H, m), δ3.80-3.67(7H, m), δ3.63-

3.40(7H, m),δ3.40-3.26(2H, m) δ2.02-2.00(6H, s)ppm. ^{13}C NMR (150 MHz, D_2O, TMS) δ175.66, 175.59, 174.65, 103.81, 103.67, 101.39, 89.28, 83.68, 82.65, 80.76, 78.20, 76.90, 76.33, 76.13, 76.02, 74.34, 73.42, 73.12, 72.38, 69.23, 68.92, 61.25, 54.97, 54.73, 23.23, 22.91 ppm. HRMS (ESI, negetive mode) calculated for $C_{28}H_{41}N_5O_{22}^{2-}$ $[M]^{2-}$ m/z 399.6127, found 399.6122.

Azido O-(β-D-glucopyranosyluronate)-(1→3)-(2-acetamido-2-deoxy-β-D –glucopyranoside)-(1→4)- (β-D-glucopyranosyluronate)-(1→3)-(2-acetamido-2-deoxy-β-D –glucopyranoside)(1→4)- (β-D-glucopyranosyluronate)-(1→3)-(2-acetamido-2-deoxy-β-D –glucopyranoside) (11)

Compound **11** was obtained from **9** (200 mg) according to Method A. (yield 109.2mg, 82%). R_f= 0.35 (n-BuOH/H_2O/EtOH/AcOH = 1/1/1/0.05)^{1}H NMR (600 MHz, D_2O, TMS) δ4.55(1H, d, *J*=8.56Hz), δ4.54(2H, dd, *J*=5.37Hz, *J*=8.41Hz), δ4.50-4.43(3H,m),δ3.93-3.87(3H,m),δ3.87-3.80(3H, m), δ3.79-3.67 (11H, m), δ4.50-4.43(3H, m), δ3.60-3.44(10H, m), δ3.37-3.28(3H, m), δ4.50-4.43(3H, m), δ2.05-1.97(m, 9H) ppm.^{13}C NMR (150 MHz, D_2O, TMS) δ174.87, 174.81, 174.78, 173.63, 173.59, 103.19, 102.88, 100.65, 100.62, 88.51, 82.90, 81.91, 79.98, 77.44, 75.89, 75.75, 75.37, 75.22, 73.57, 72.63, 72.32, 71.56, 68.46, 68.35, 68.15, 22.45, 22.15 ppm. HRMS (ESI, negetive mode) calcd for $C_{42}H_{61}N_6O_3^{3-}$ $[M+1H]^{3-}$ m/z 392.4432, found 392.7766.

Methyl O-(methyl 2,3,4-tri-O-acetyl-β-D-glucopyranosyluronate)-(1→3)-(4,6-di-O-acetyl-2-acetamido-2-deoxy-β-D-glucopyranoside)-(1→4)-(methyl 2,3-di-O-acetyl-β-D-glucopyranosyluronate)-(1→3)-4,6-di-O-acetyl-2-acetamido-2-deoxy-D-glucopyranoside (12) and Methyl O-(methyl 2,3,4-tri-O-acetyl-β-D-glucopyranosyluronate)-(1→3)-(4,6-di-O-acetyl-2-acetamido-2-deoxy-β-D-glucopyranoside)-(1→4)-(methyl 2,3-di-O-acetyl-β-D-glucopyranosyluronate)-(1→3)-(4,6-di-O-acetyl-2-acetamido-2-deoxy-D-glucopyranoside)-(1→4)-(methyl 2,3-di-O-acetyl-β-D-glucopyranosyluronate)-(1→3)-4,6-di-O-acetyl-2-acetamido-2-deoxy-D-glucopyranoside (13)

HA Enzymatic degradation mixture (3.3g) was treated with HCl/MeOH (0.5 M,

330 mL) at 0ºC, after stirring for 1h, it was naturally raised to rt and stired for 24h, monitored by TLC until complete reaction and then the solution was neutralized by NaOH, concentrated under reduced pressure to obtain white solid. The solid was washed by 50mL DMF, and then the solvent was removed under reduced pressure to obtain white solid. The solid was dissolved in 30mL DCM, 179mg DMAP, 9mL Et_3N were added, 30mL Ac_2O was added dropwise under ice bath, and it was naturally warmed to rt. After the reaction was completed, water was added to quench under ice bath, and the organic phase was separated, washed with saturated aqueous $NaHCO_3$, and saturated brine, dried over anhydrous Na_2SO_4, and concentrated. Through silica gel chromatography separation (DCM/ EtOAc/ MeOH=1/1/0.06 to1/1/0.08, containing 0.1%Et_3N), 0.8g **12** was obtained as white solid) and 0.7g **13** as white solid. **12**: R_f= 0.32 (DCM/ EA/ MeOH= 1: 1: 0.2) and **13** R_f= 0.27 (DCM/ EA/ MeOH= 1: 1: 0.2).

Compound 12: ^{1}H NMR(400MHz, $CDCl_3$, TMS) δ5.99(2H, q, *J*= 7.03Hz), δ5.37(1H, d, *J*= 3.04Hz), δ5.31(1H, d, *J*=3.11Hz), δ5.16(2H, m), δ5.04(1H, t, *J*=8.73Hz), δ4.95-4.80(3H, m), δ4.73(1H, d, *J*=8.22Hz), δ4.69(1H, d, *J*=7.18Hz), δ4.66(1H, d, *J*= 7.83Hz), δ4.59(1H, dd, *J*= 3.04Hz, *J*= 10.87Hz), δ4.53(1H, dd, *J*= 3.23Hz, *J*= 10.93Hz), δ4.17-3.92(7H, m), δ3.88-3.70(8H, m), δ3.48(3H, s), δ3.33(1H,m), δ2.09-1.90 (33H, m)ppm. ^{13}C NMR (100 MHz, $CDCl_3$, TMS) δ171.12, 171.02, 170.57, 169.98, 169.90, 169.74, 169.34, 167.81, 167.01, 100.33, 100.01, 99.44, 99.10, 75.35, 74.69, 74.09, 73.89, 72.49, 72.17, 72.09, 71.26, 71.18, 70.91, 70.80, 69.01, 68.48, 67.86, 62.56, 61.76, 56.90, 54.51, 53.07, 52.82, 23.56, 23.46, 20.67, 20.55, 20.51, 20.42 ppm.

Compound 13: ^{1}H NMR(600MHz, $CDCl_3$, TMS) δ5.90(2H, d, *J*=21.81Hz), δ5.36(1H, s), δ5.30(2H, d, *J*=9.25Hz), δ5.20-5.10(2H, m), δ5.07-4.97 (2H, m), δ4.90(1H, t, *J*= 7.75Hz), δ4.87-4.77(3H, m), δ4.74(1H, d, *J*=7.75Hz), δ4.70(1H, d, *J*=8.10Hz), δ4.68-4.57(4H, m), δ4.50(2H, d, *J*=9.51Hz), δ4.17-3.92(12H, m), δ3.87-3.66(13H, m), δ3.49-3.44(3H, s), δ3.40-3.19(2H, m)ppm. ^{13}C NMR (100 MHz, $CDCl_3$, TMS) δ171.12, 171.09, 171.02, 170.58, 170.13, 169.97, 169.85, 169.39,

169.32, 169.25, 167.85, 167.20, 162.62, 100.26, 100,10, 100,01, 99.45, 99.21, 98.76, 75.37, 75.24, 74.67, 74.18, 74.08, 73.99, 72.50, 72.47, 72.19, 72.09, 71.23, 71.20, 71.15, 71.02, 70.93, 70.73, 69.02, 68.46, 68.32, 67.90, 62.55, 61.77, 57.02, 54.72, 54.42, 53.98, 53.15, 53.12, 52.94, 23.64, 23.54, 23.52, 20.72, 20.66, 20.60, 20.54, 20.47 ppm.

Methyl O-(β-D-glucopyranosyluronate)-(1→3)-(2-acetamido-2-deoxy-β-D –glucopyranoside)-(1→4)- (β-D-glucopyranosyluronate)-(1→3)-2-acetamido-2-deoxy-β-D –glucopyranoside (14)

Compound **14** was obtained from **12** (200 mg) according to Method A. (yield 116mg, 85%). R_f= 0.40 (n-BuOH/H_2O/EtOH/AcOH = 1/1/1/0.05)^{1}H NMR(400MHz, D_2O, TMS) δ4.50-4.32(4H, m), δ3.89-3.54(12H, m), δ3.54-3.34(8H, m), δ3.34-3.19(3H, m), δ1.97-1.89(9H, m)ppm. ^{13}C NMR (100 MHz, D_2O, TMS) δ175.45, 174.87, 174.73, 174.13, 103.06, 102.91, 101.69, 100.52, 82.89, 76.31, 75.70, 75.33, 75.24, 73.53, 72.64, 72.37, 71.65, 68.56, 68.44, 60.60, 60.44, 57.08, 54.30, 54.18, 23.21, 22.44, 22.35, 22.16 ppm. HRMS (ESI, negetive mode) calculated for $C_{29}H_{44}N_2O_{23}^{2-}$ $[M]^{2-}$ m/z 394.1173, found 394.1170.

Methyl O-(β-D-glucopyranosyluronate)-(1→3)-(2-acetamido-2-deoxy-β-D –glucopyranoside)-(1→4)- (β-D-glucopyranosyluronate)-(1→3)-(2-acetamido-2-deoxy-β-D –glucopyranoside)(1→4)- (β-D-glucopyranosyluronate)-(1→3)-(2-acetamido-2-deoxy-β-D –glucopyranoside) (15)

Compound **15** was obtained from **13** (200 mg) according to Method A. (yield 108.8mg, 82%). R_f = 0.32 (n-BuOH/H_2O/EtOH/AcOH = 1/1/1/0.05)^{1}H NMR(600MHz, D_2O, TMS) δ4.54(2H, dd, *J*=6.00Hz, *J*=8.52Hz), δ4.46(3H, d, *J*=7.65Hz), δ4.44(1H, d, *J*=8.44Hz), δ3.93-3.88(3H, m), δ3.85-3.79(3H, m), δ3.78-3.68(11H, m), δ3.59-3.54(2H, m), δ3.54-3.44(11H, m), δ3.36-3.29(3H, m), δ2.02-1.99(9H, m)ppm. ^{13}C NMR (150 MHz, D_2O, TMS) δ175.73, 175.66, 175.52, 174.50, 103.85, 102.49, 101.39, 101.36, 83.71, 83.18, 83.13, 80.74, 80.69, 76.77, 76.65, 76.24, 76.15, 76.13, 76.09, 76.01, 74.35, 74.32, 72.37, 69.36, 69.23, 69.12, 61.43, 61.26, 61.24, 57.86, 55.09, 55.04, 54.9

7, 23.22, 22.95 ppm. HRMS (ESI, negetive mode) calcd for $C_{43}H_{64}N_3O_{34}^{3-}$ $[M]^{3-}$ m/z 388.7796, found 388.7788.

Figure S1 ^{1}H NMR, ^{13}C NMR HRMS and HPLC spectra of the compounds **2-15**

O-(Methyl 2, 3, 4-tri-O-acetyl-β-D-glucopyranosyluronate)-(1→3)-1, 4, 6-tri-O-acetyl-2-deoxy-2-acetamido-D- glucopyranose (2)

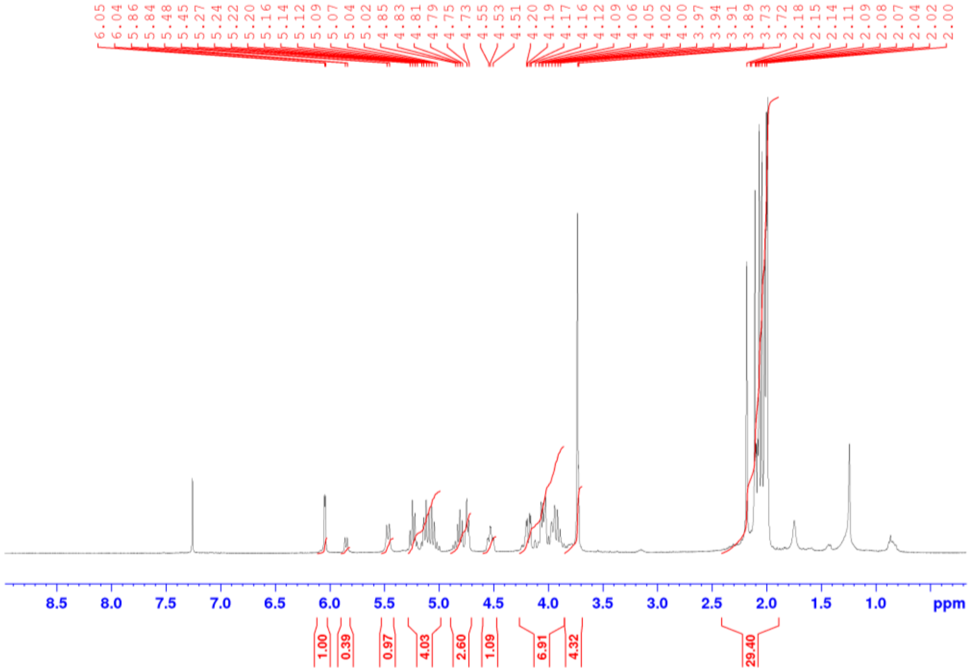

Figure S1.1.(1) ^{1}H NMR of the compound 2 (400MHz, $CDCl_3$, TMS)

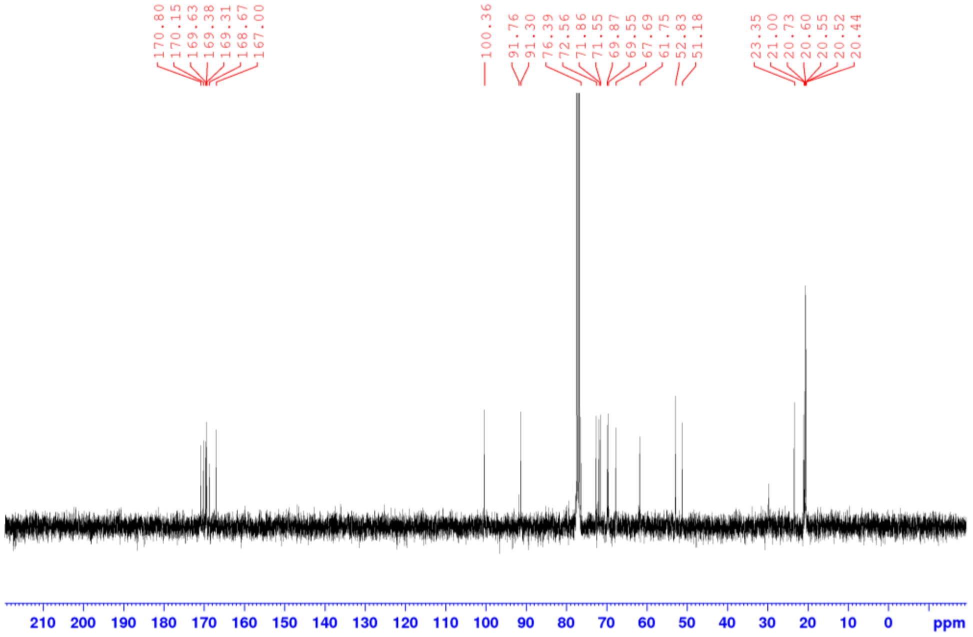

Figure S1.1.(2) ^{13}C NMR of the compound 2 (100MHz, $CDCl_3$, TMS)

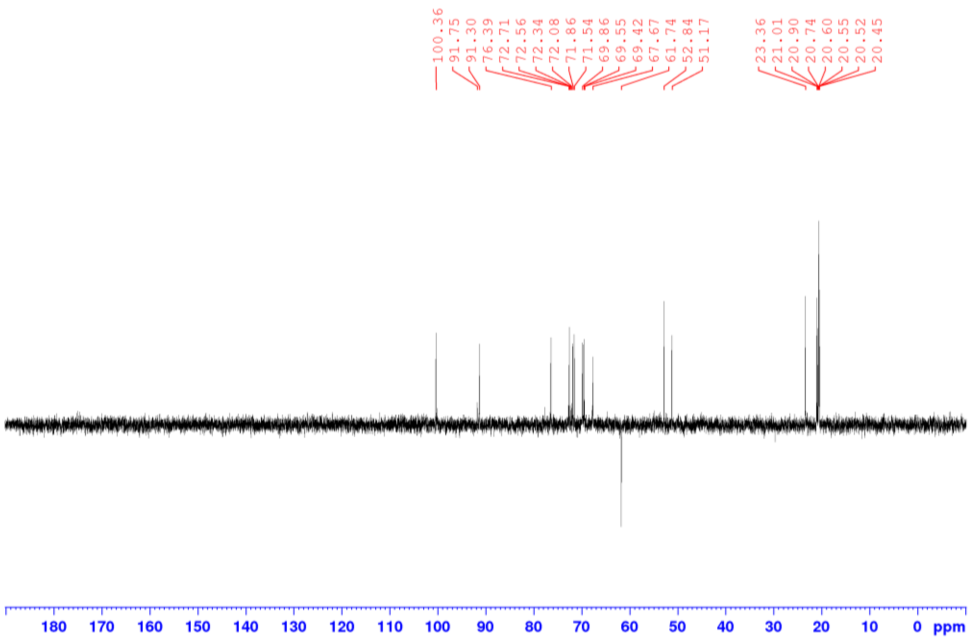

Figure S1.1.(3) DEPT-135 NMR of the compound 2 (100MHz, $CDCl_3$, TMS)

O-(Methyl 2, 3, 4-tri-O-acetyl-β-D-glucopyranosyluronate)-(1→3)-2-methyl-(4, 6di-O-acetyl-1, 2-dideoxy-α-D glucopyranose)[2, 1-d] 2-oxazoline(3)

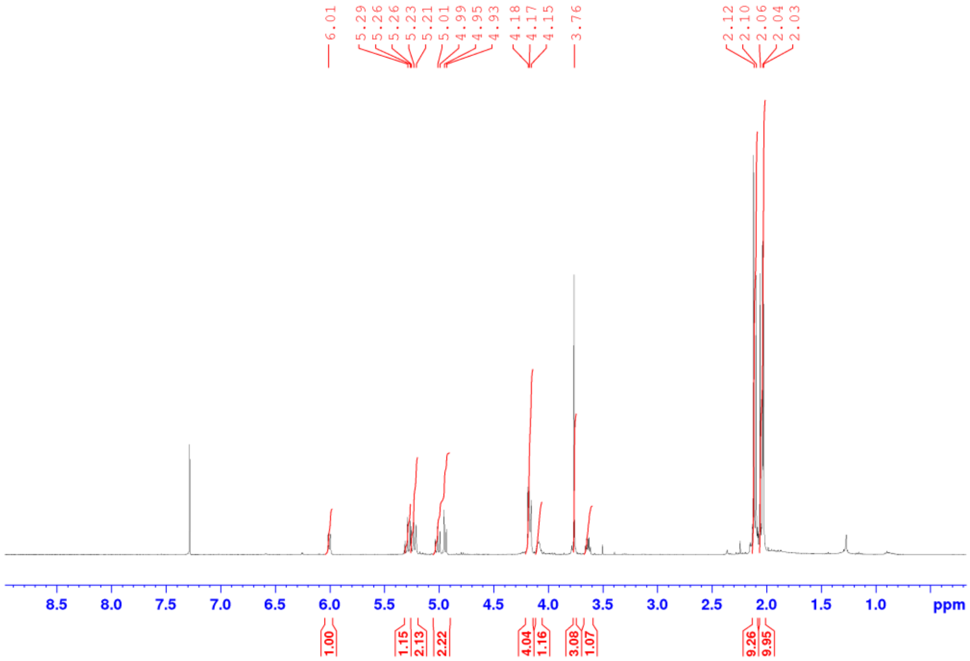

Figure S1.2.(1) ^{1}H NMR of the compound 3 (400MHz, $CDCl_3$, TMS)

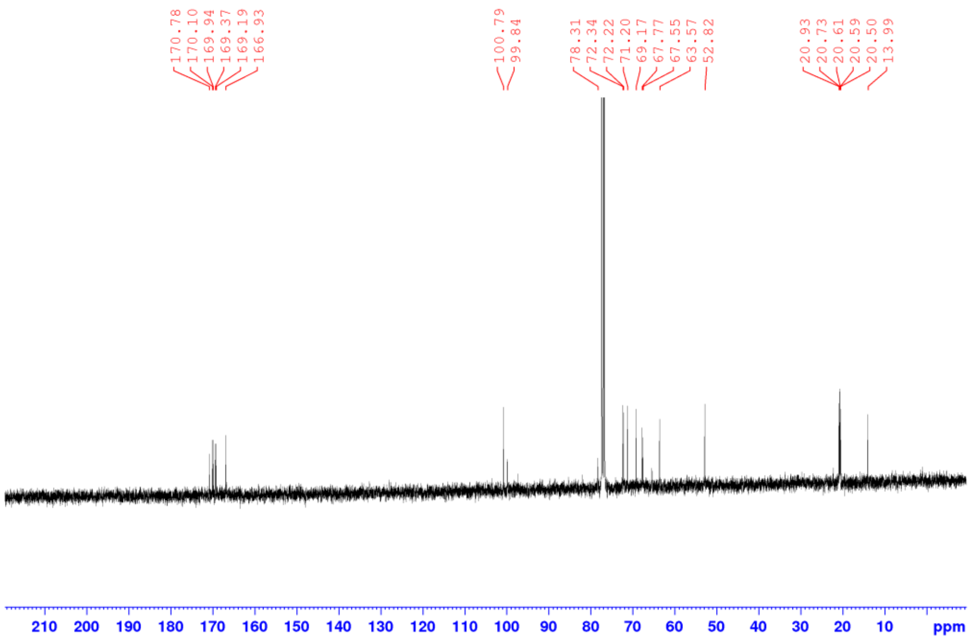

Figure S1.2.(2) ^{13}C NMR of the compound 3 (100MHz, $CDCl_3$, TMS)

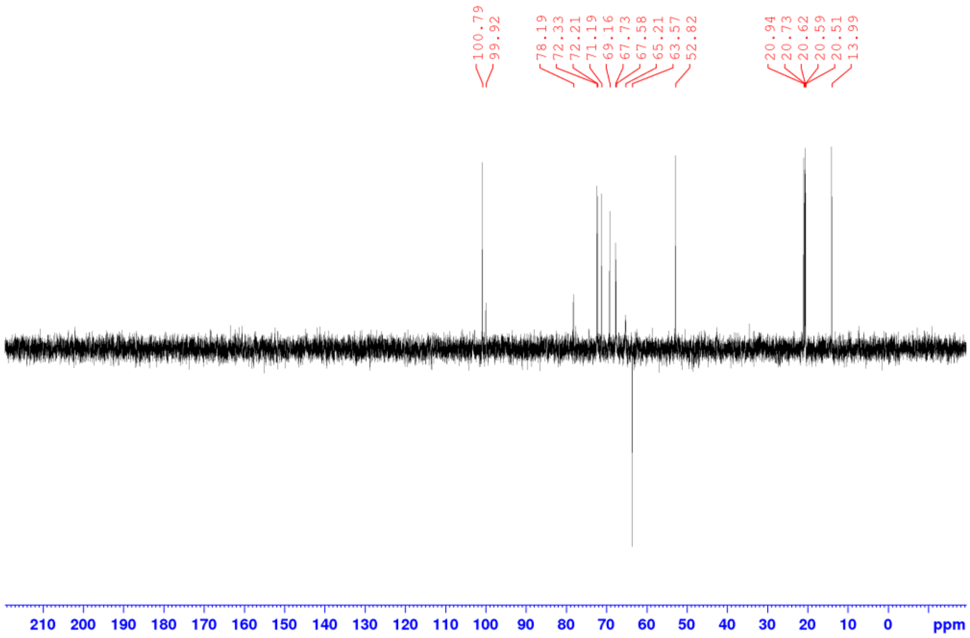

Figure S1.2.(3) DEPT-135 NMR of the compound 3 (100MHz, $CDCl_3$, TMS)

Azido O-(Methyl 2, 3, 4-tri-O-acetyl-β-D-glucopyranosyluronate)-(1→3)-2-methyl-(4, 6di-O-acetyl-1, 2-dideoxy-α-D glucopyranose) (4)

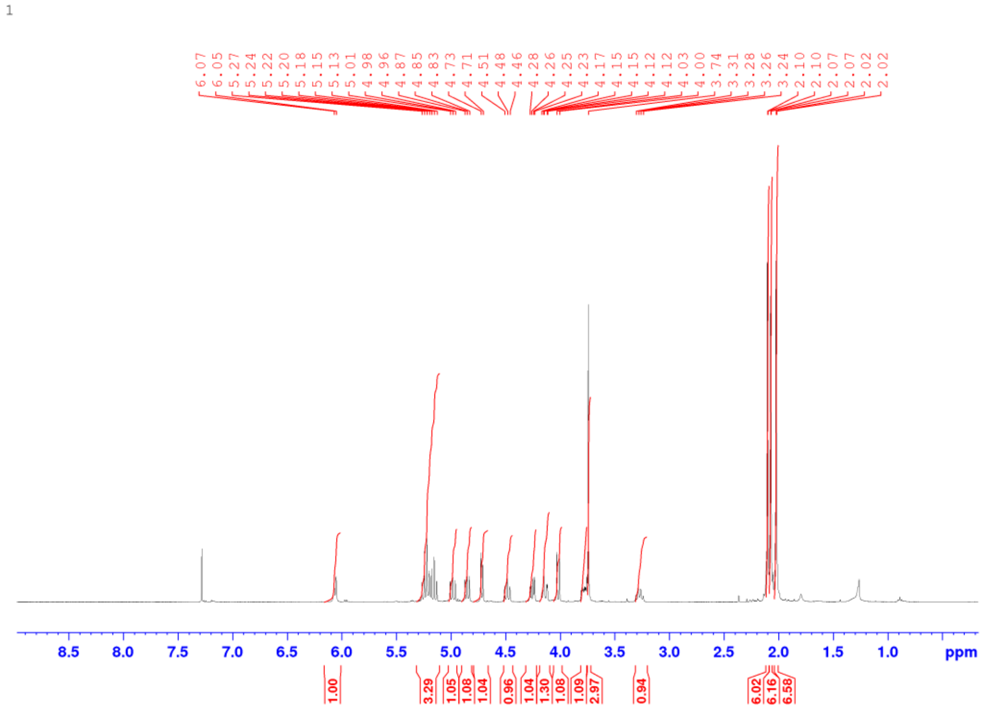

Figure S1.3.(1) ^{1}H NMR of the compound 4 (400MHz, $CDCl_3$, TMS)

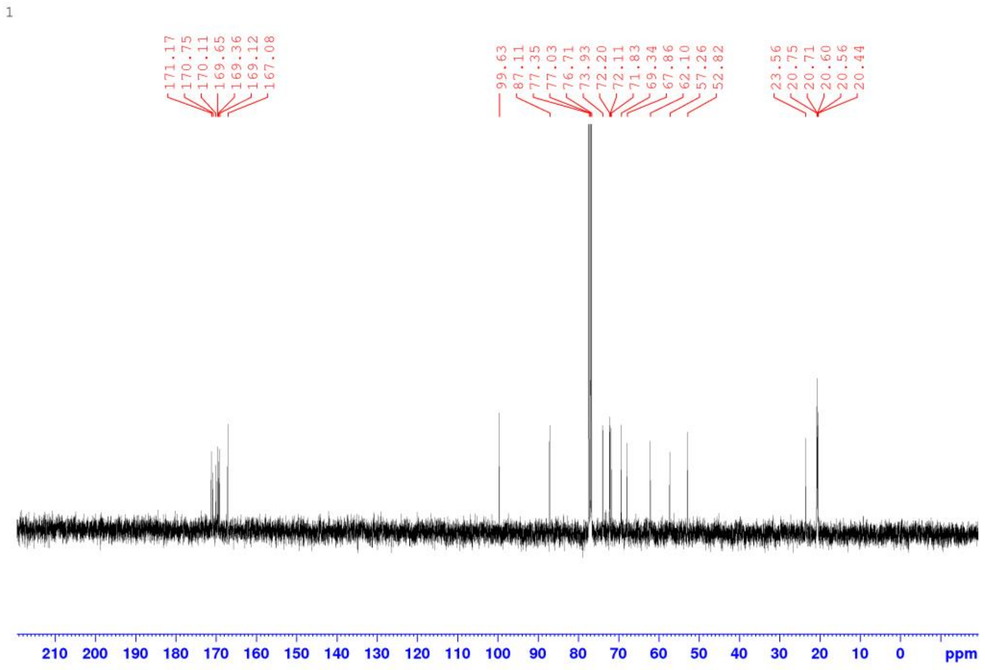

Figure 1.3.(2) ^{13}C NMR of the compound 4 (100MHz, $CDCl_3$, TMS)

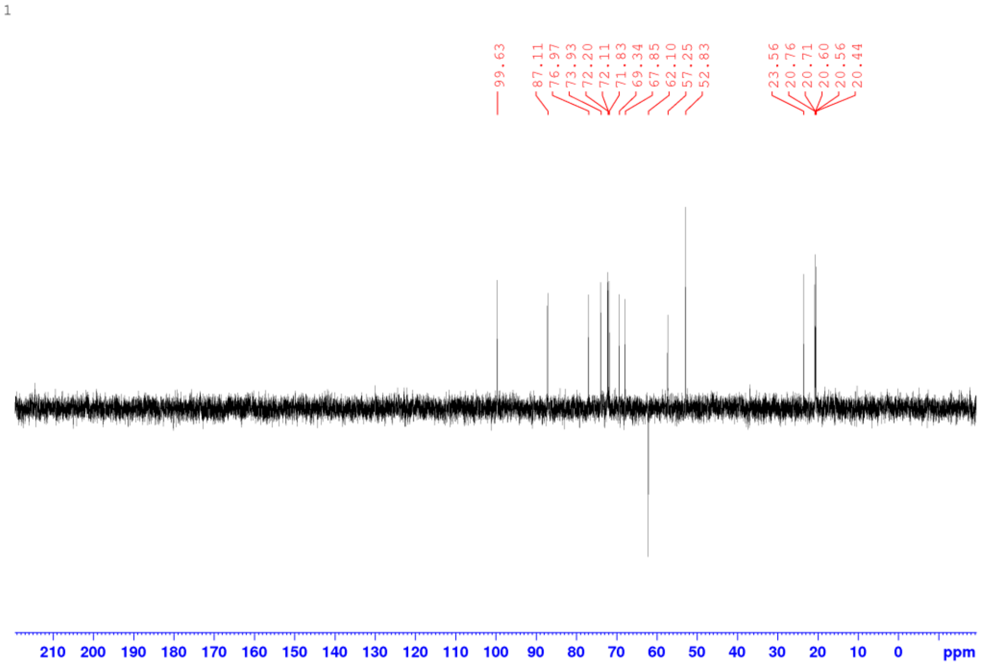

Figure S1.3.(3) DEPT-135 NMR of the compound 5 (100MHz, $CDCl_3$, TMS)

Methyl O-(Methyl 2, 3, 4-tri-O-acetyl-β-D-glucopyranosyluronate)-(1→3)-2-methyl-(4, 6di-O-acetyl-1, 2-dideoxy-α-D glucopyranose) (5)

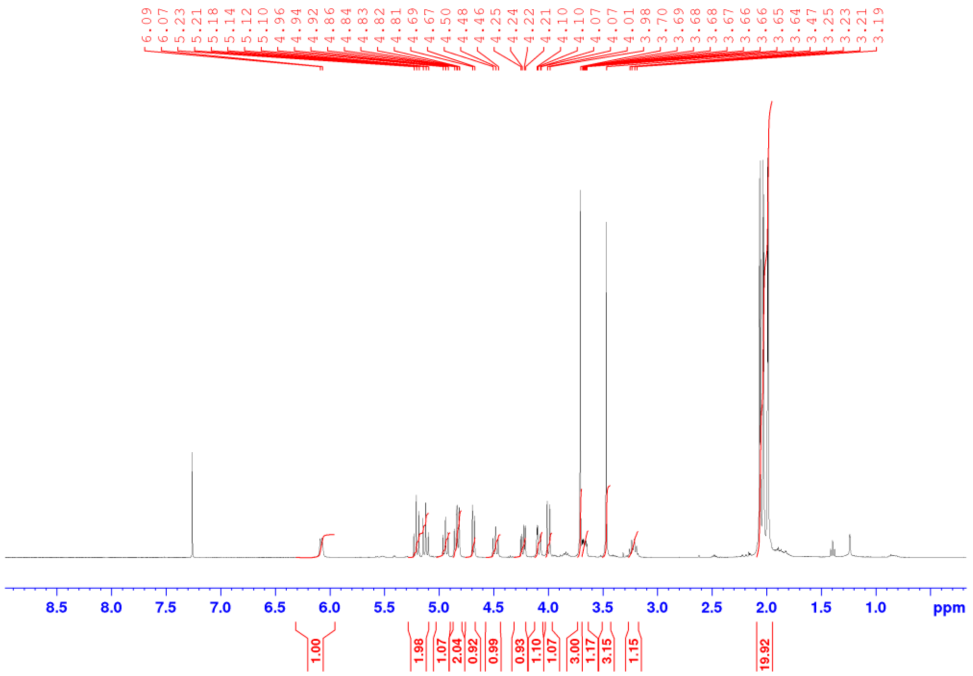

Figure S1.4.(1) ^{1}H NMR of the compound 5 (400MHz, $CDCl_3$, TMS)

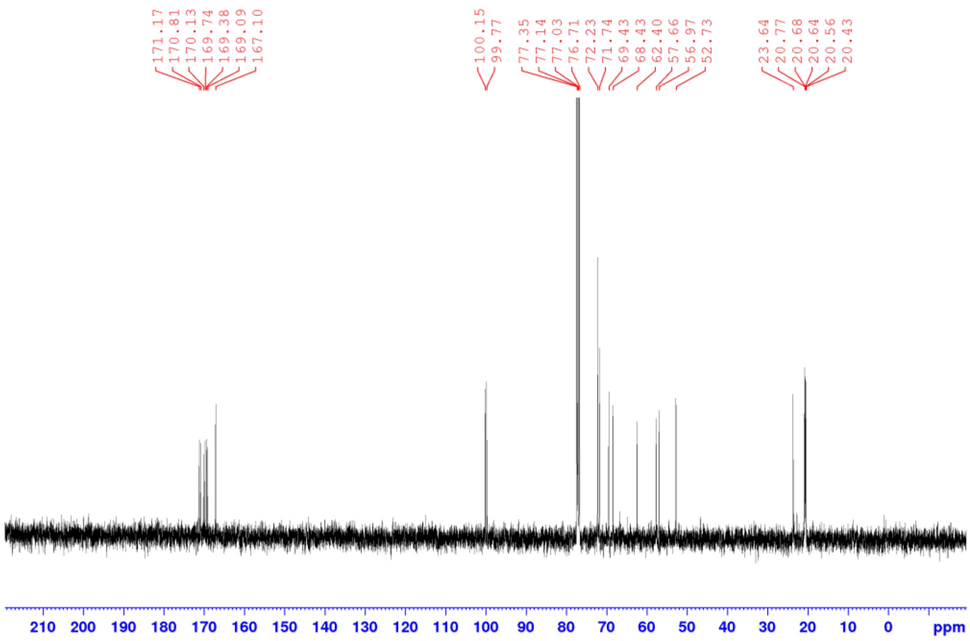

Figure S1.4.(2) ^{13}C NMR of the compound 5 (100MHz, $CDCl_3$, TMS)

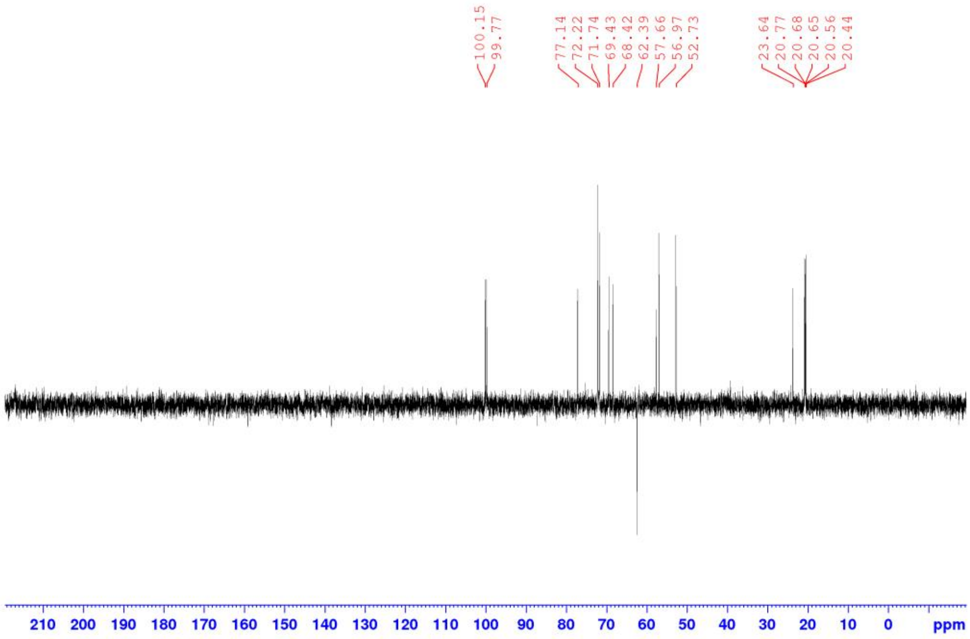

Figure S1.4.(3) DEPT-135 NMR of the compound 5 (100MHz, $CDCl_3$, TMS)

Azido O-(β-D-glucopyranosyluronate)-(1→3)-2-acetamido-2-deoxy-β-D-glucopyranoside (6)

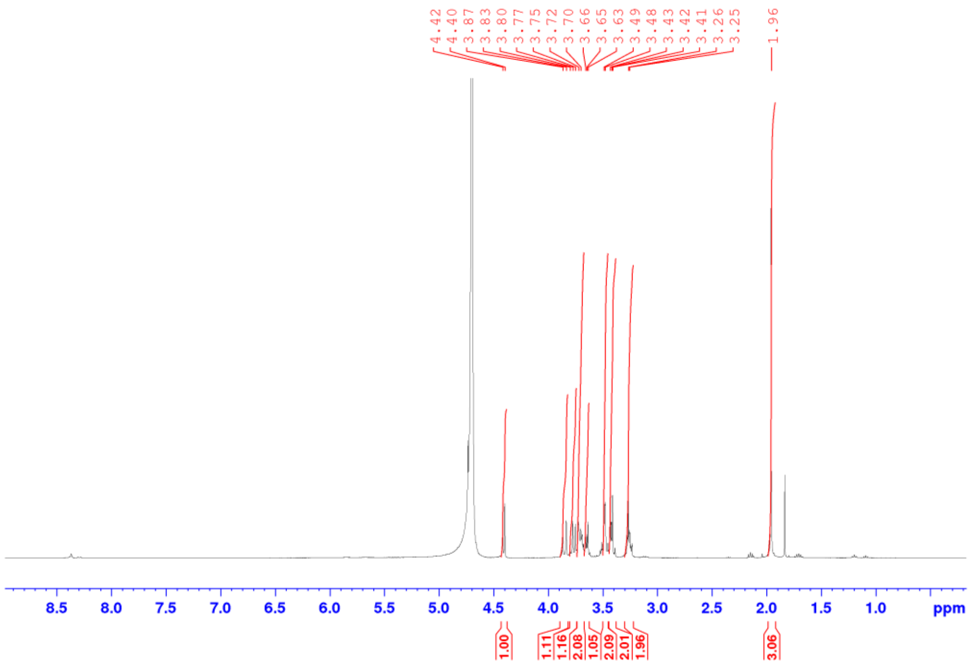

Figure S1.5.(1) ^{1}H NMR of the compound 6 (400MHz, D_2O, TMS)

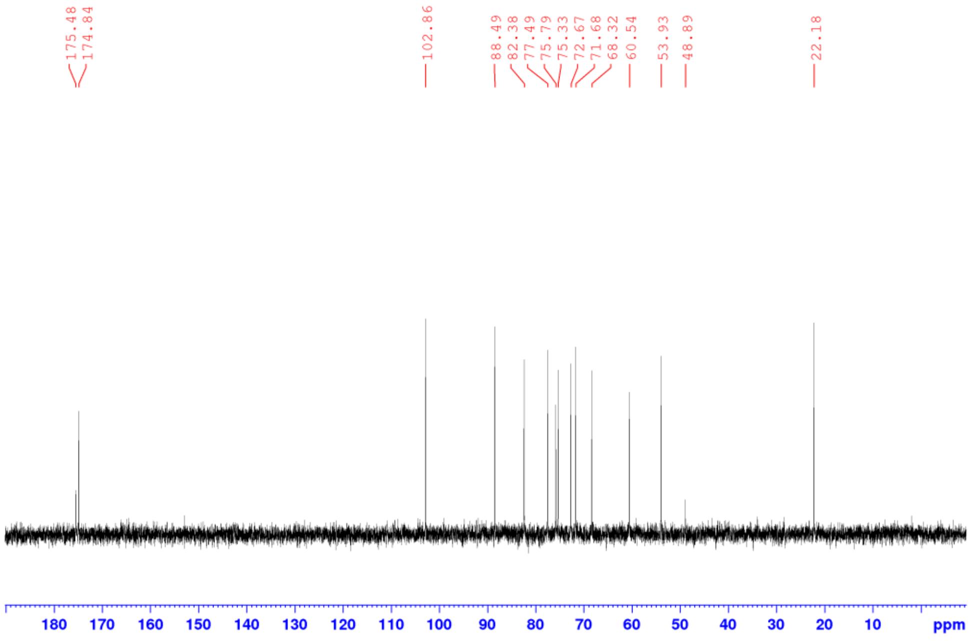

Figure S1.5.(2) ^{13}C NMR of the compound 6 (100MHz, D_2O, TMS)

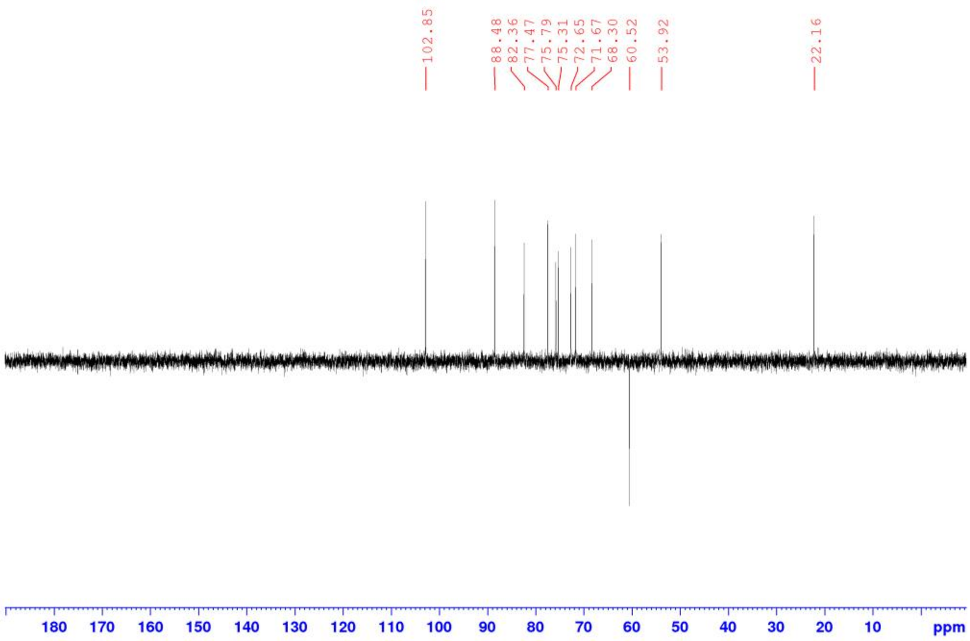

Figure S1.5.(3) DEPT-135 NMR of the compound 6 (100MHz, D_2O, TMS)

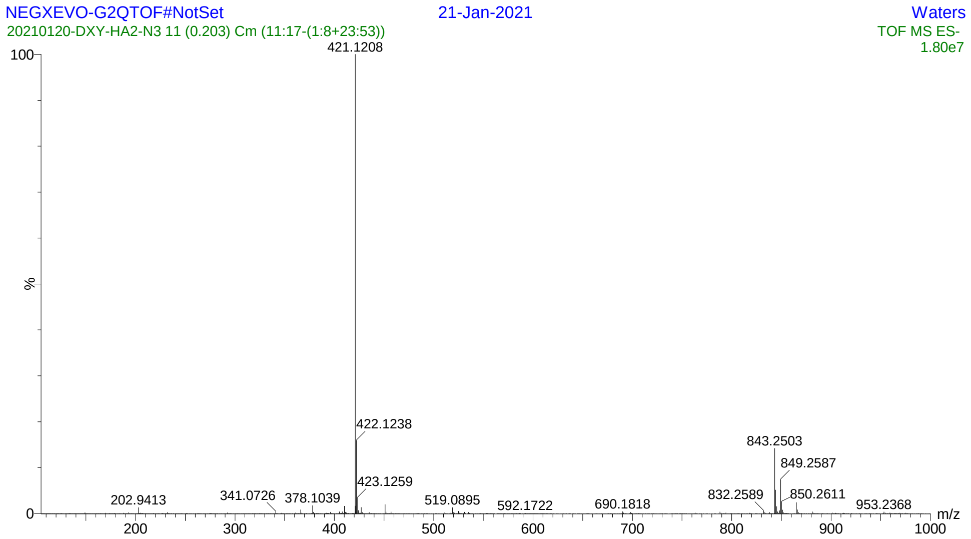

Figure S1.5.(4) HR-MS of the compound 6

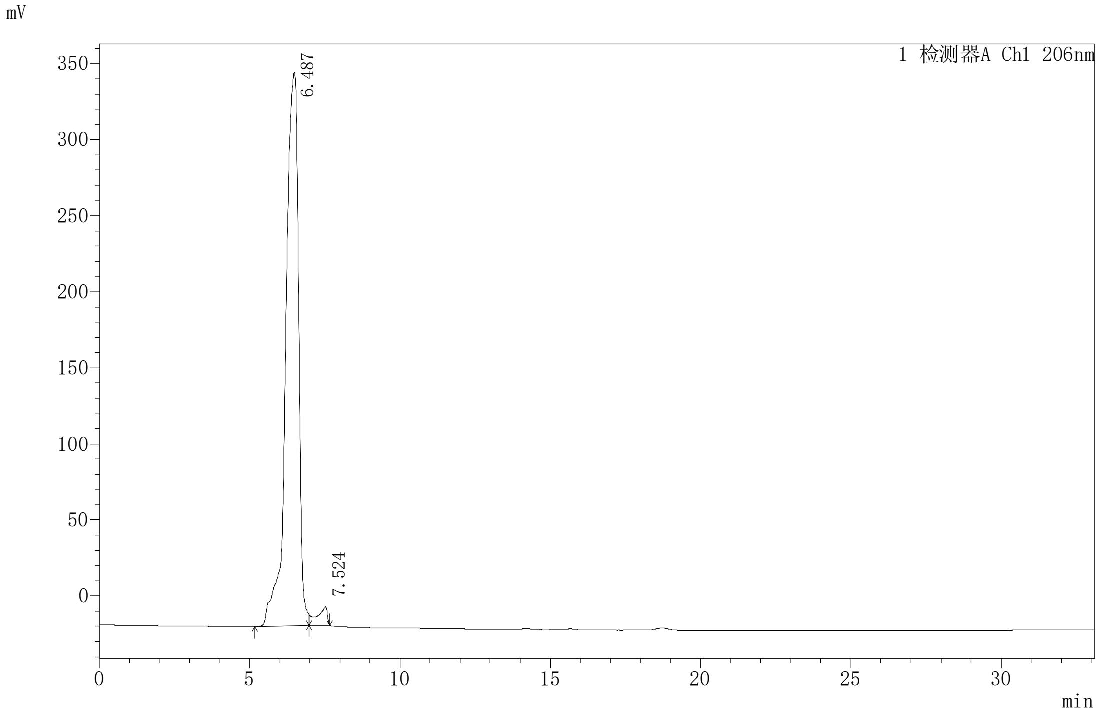

Figure S1.5.(5) HPLC of compound 6

Table S1.1 HPLC of compound 6

Peak	Residual time(Min)	Area	Height	Concentration（%）	Area%
1	6.487	10768094	364105	97.370	97.370
2	7.524	290845	12329	2.630	2.630
		11058939		100.000	100.000

Methyl O-(β-D-glucopyranosyluronate)-(1→3)-2-acetamido-2-deoxy-β-D – glucopyranoside (7)

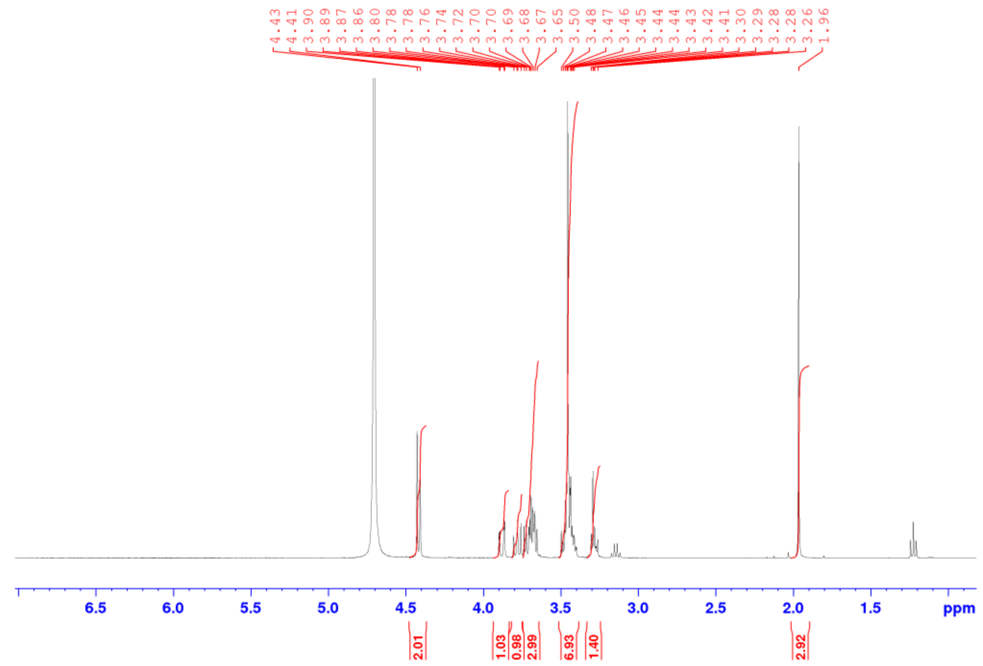

Figure S1.6.(1) ^{1}H NMR of the compound 7(400MHz, D_2O, TMS)

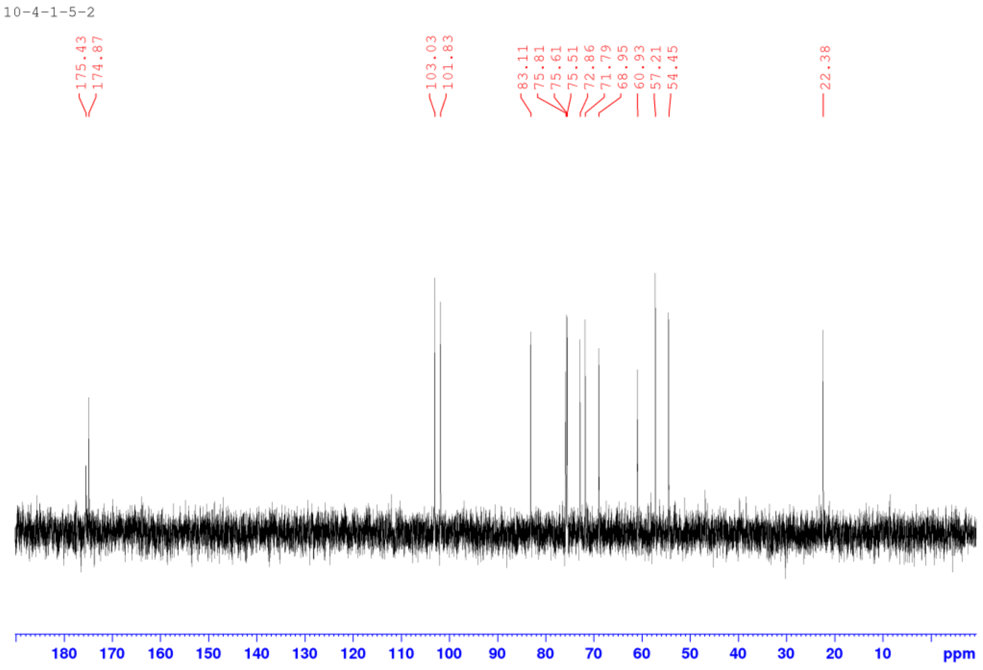

Figure S1.6.(2) ^{13}C NMR of the compound 7 (100MHz, D_2O, TMS)

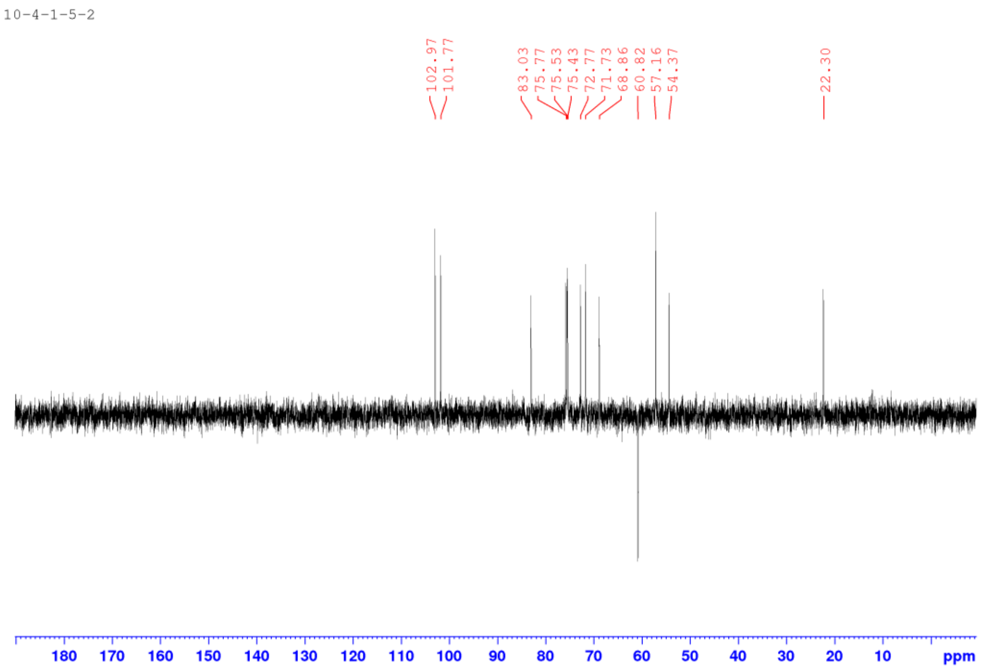

Figure S1.6.(3) DEPT-135 NMR of the compound 7 (100MHz, D_2O, TMS)

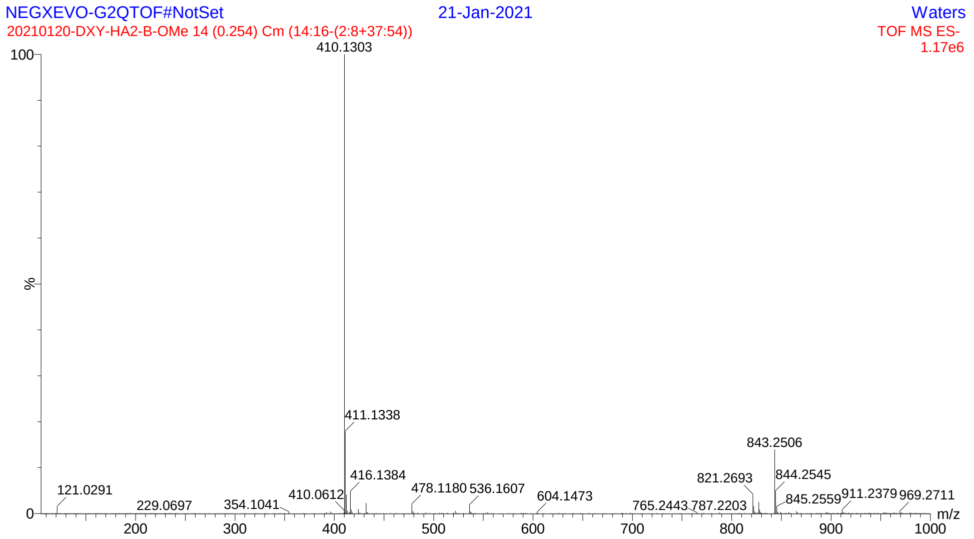

Figure S1.6.(4) HR-MS of the compound 7

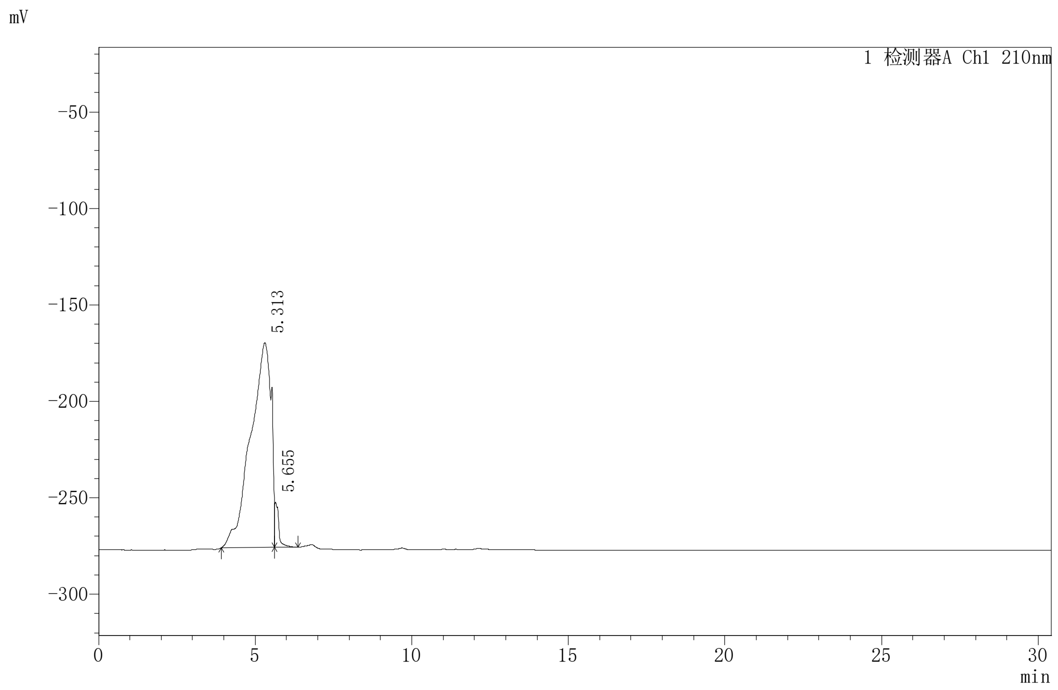

Figure S1.6.(5) HPLC of compound 7

Table S1.2 HPLC of compound 7

Peak	Residual time(Min)	Area	Height	Concentration（%）	Area%
1	5.313	4832090	105953	96.049	96.049
2	5.655	198775	24197	3.951	3.951
		5030865		100.000	100.000

Azido O-(methyl 2,3,4-tri-O-acetyl-β-D-glucopyranosyluronate)-(1→3)-(4,6-di-O-acetyl-2-acetamido-2-deoxy-β-D-glucopyranoside)-(1→4)-(methyl 2,3-di-O-acetyl-β-D-glucopyranosyluronate)-(1→3)-4,6-di-O-acetyl-2-acetamido-2-deoxy-D-glucopyranoside (8)

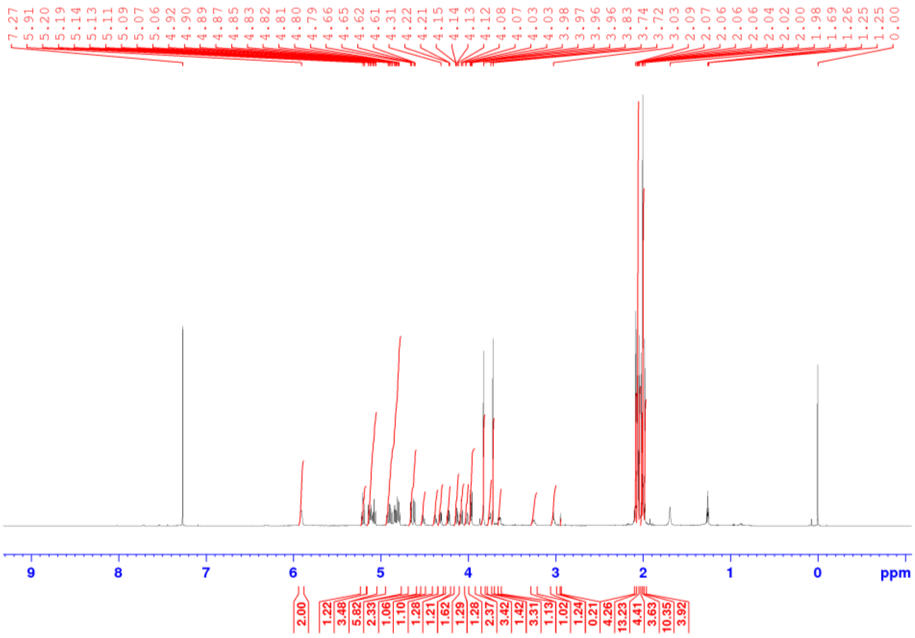

Figure S1.7.(1) 1H NMR of the compound 8 (600MHz, CDCl3, TMS)

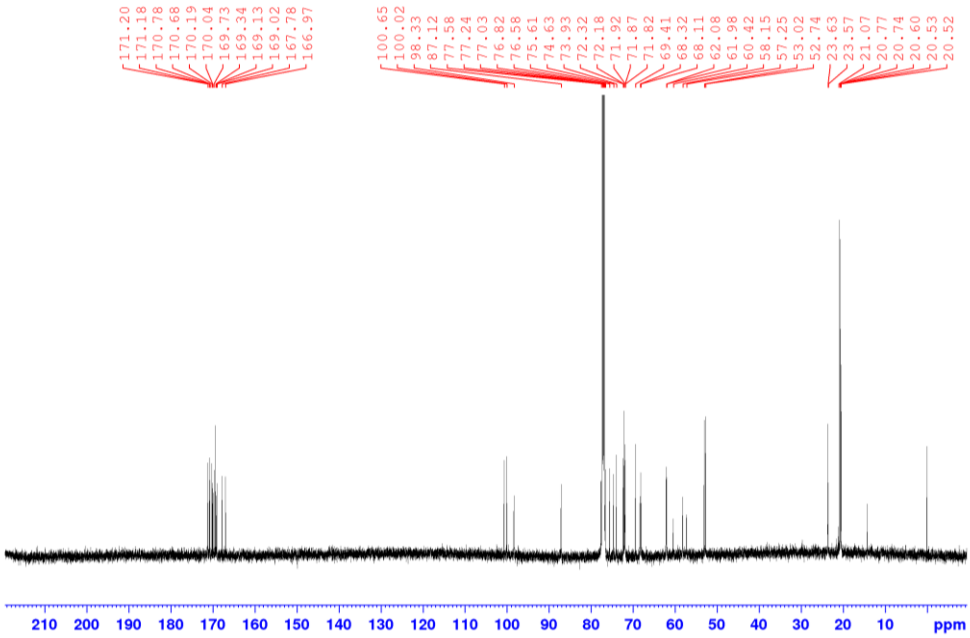

Figure S1.7.(2) ^{13}C NMR of the compound 8 (150MHz, $CDCl_3$, TMS)

Azido O-(methyl 2,3,4-tri-O-acetyl-β-D-glucopyranosyluronate)-(1→3)-(4,6-di-O-acetyl-2-acetamido-2-deoxy-β-D-glucopyranoside)-(1→4)-(methyl 2,3-di-O-acetyl-β-D-glucopyranosyluronate)-(1→3)-(4,6-di-O-acetyl-2-acetamido-2-deoxy-D-glucopyranoside)-(1→4)-(methyl 2,3-di-O-acetyl-β-D-glucopyranosyluronate)-(1→3)-4,6-di-O-acetyl-2-acetamido-2-deoxy-D-glucopyranoside (9)

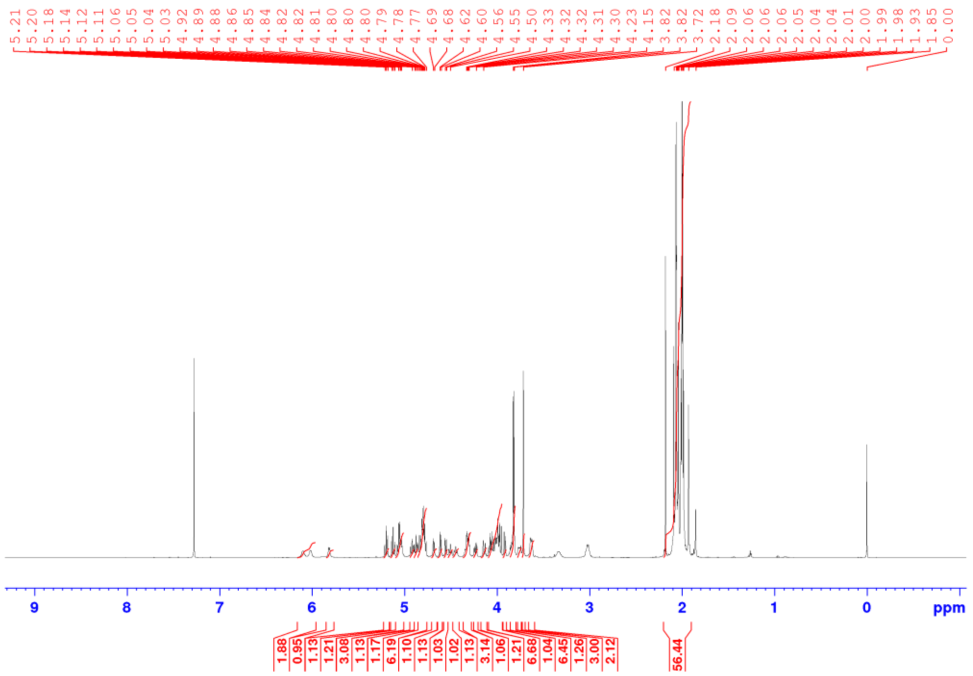

Figure S1.8.(1) ^{1}H NMR of the compound 9 (600MHz, $CDCl_3$, TMS)

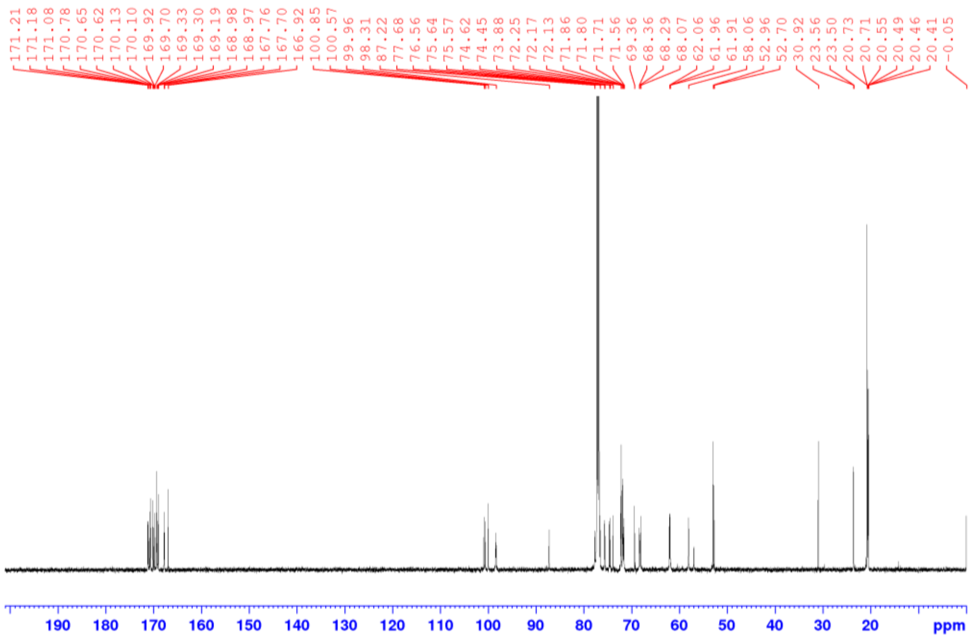

Figure S1.8.(2) ^{13}C NMR of the compound 9 (150MHz, $CDCl_3$, TMS)

Azido O-(β-D-glucopyranosyluronate)-(1→3)-(2-acetamido-2-deoxy-β-D –glucopyranoside)-(1→4)- (β-D-glucopyranosyluronate)-(1→3)-2-acetamido-2-deoxy-β-D –glucopyranoside (10)

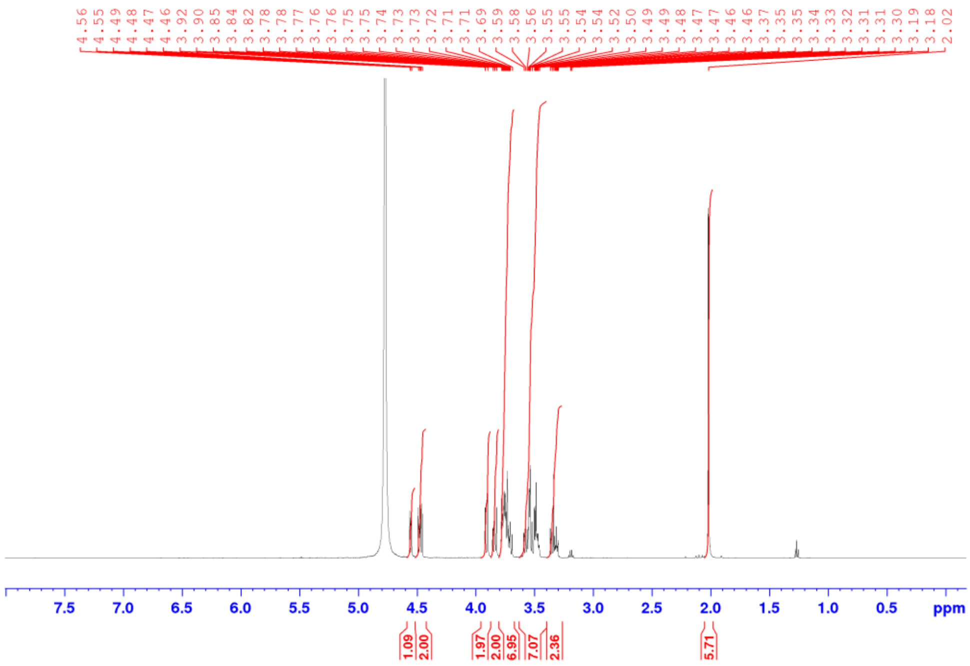

Figure S1.9.(1) ^{1}H NMR of the compound 10 (600MHz, D_2O, TMS)

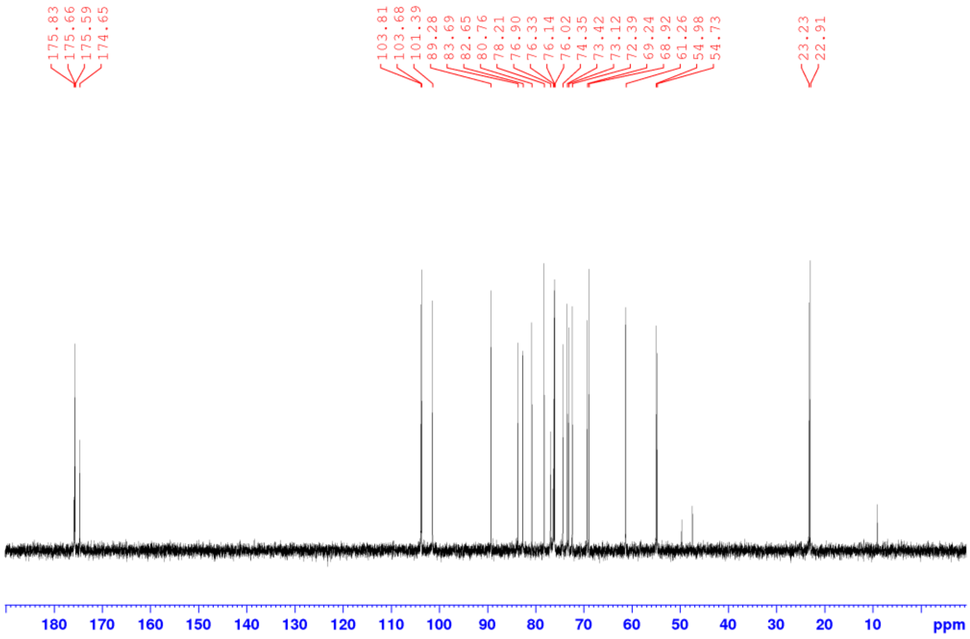

Figure S1.9.(2) ^{13}C NMR of the compound 10 (150MHz, D_2O, TMS)

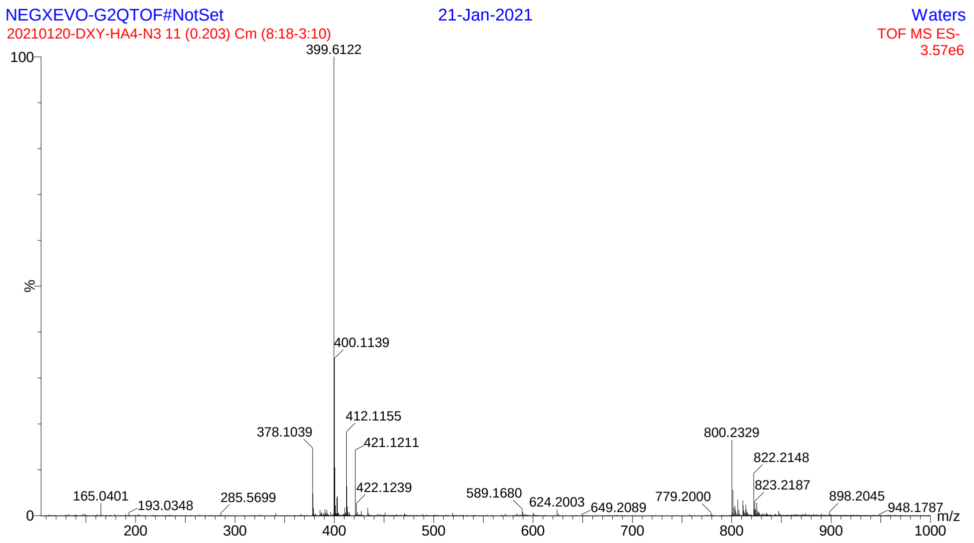

Figure S1.9.(3) HR-MS of the compound 10

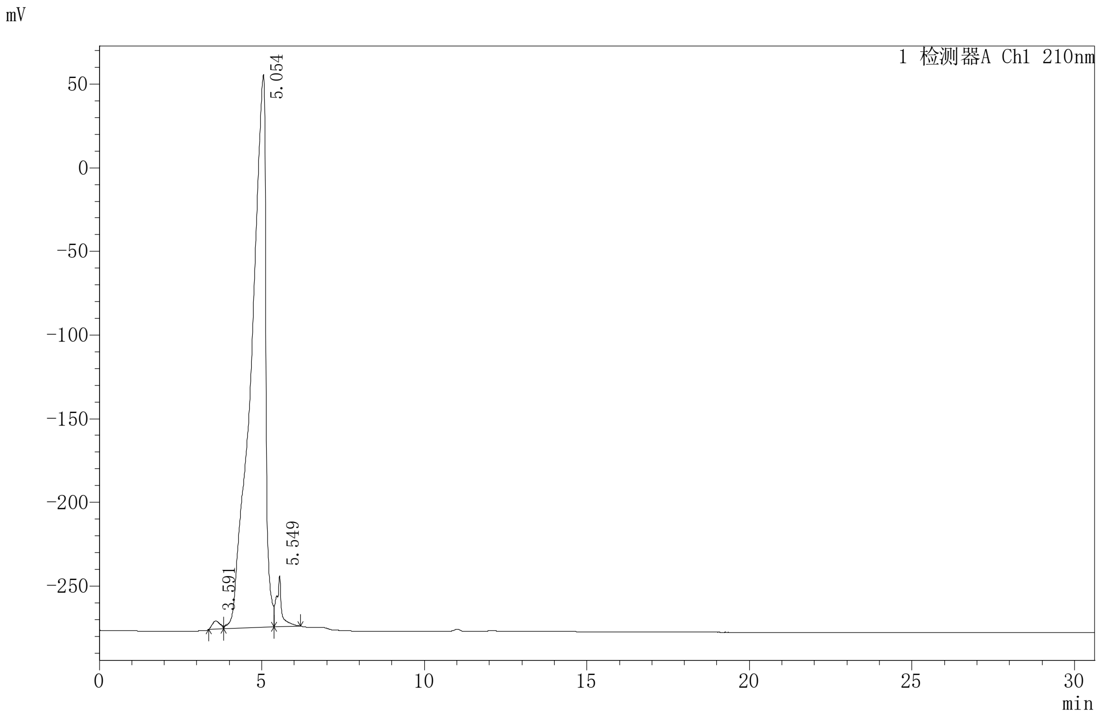

Figure S S1.9.(4) HPLC of compound 10

Table S1.3 HPLC of compound 10

Peak	Residual time(Min)	Area	Height	Concentration (%)	Area%
1	3.591	77853	4740	0.729	0.729
2	5.054	10261038	330596	96.079	96.079
3	5.549	340898	30530	3.192	3.192
		10679790		100.000	100.000

Azido O-(β-D-glucopyranosyluronate)-(1→3)-(2-acetamido-2-deoxy-β-D –glucopyranoside)-(1→4)- (β-D-glucopyranosyluronate)-(1→3)-(2-acetamido-2-deoxy-β-D –glucopyranoside)(1→4)- (β-D-glucopyranosyluronate)-(1→3)-(2-acetamido-2-deoxy-β-D –glucopyranoside) (11)

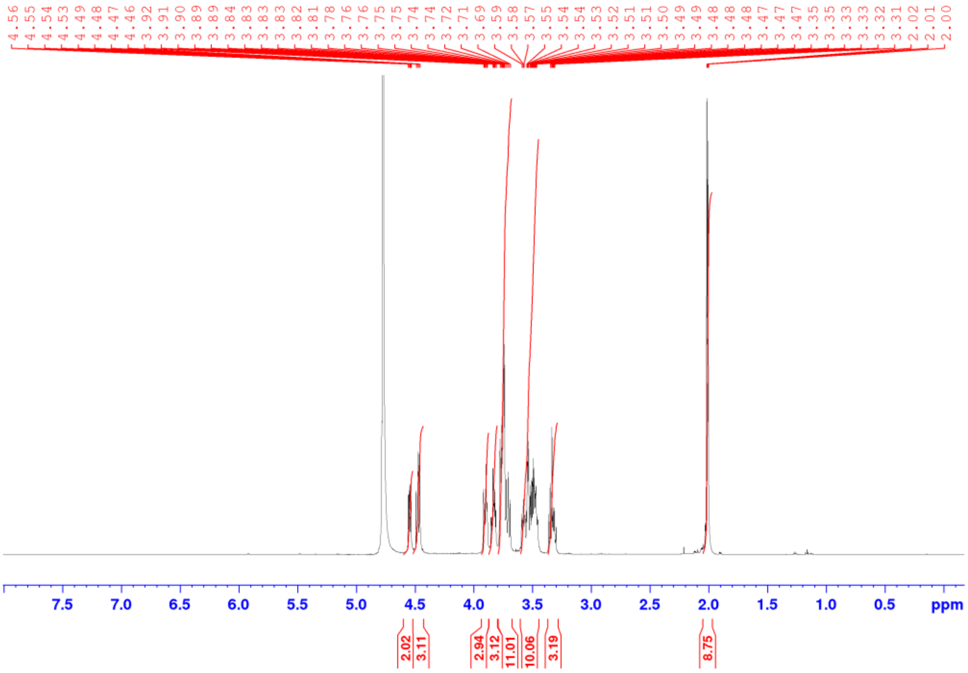

Figure S1.10.(1) ^{1}H NMR of the compound 11 (600MHz, D_2O, TMS)

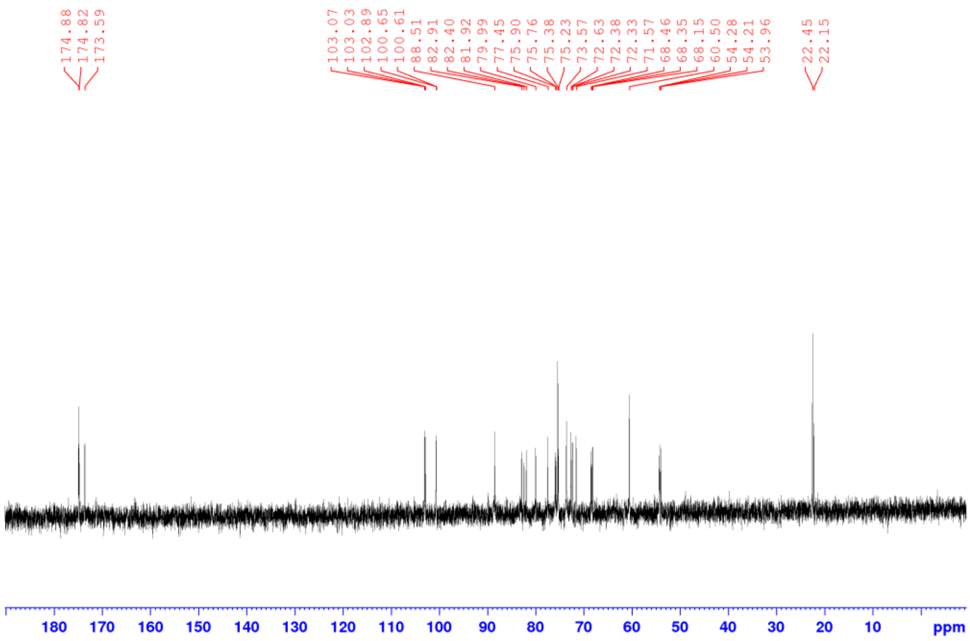

Figure S1.10.(2) ^{13}C NMR of the compound 11 (1500MHz, D_2O, TMS)

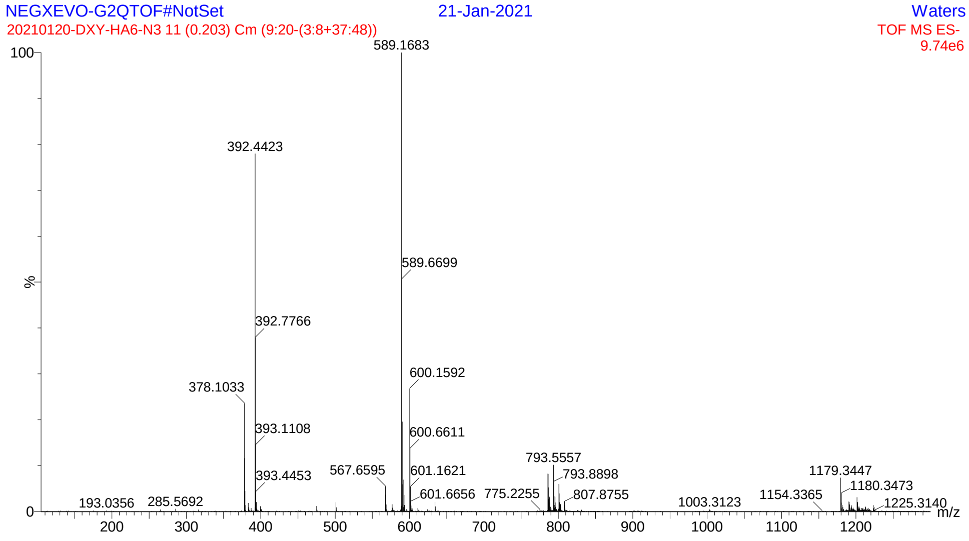

Figure S1.10.(3) HR-MS data of the compound 11

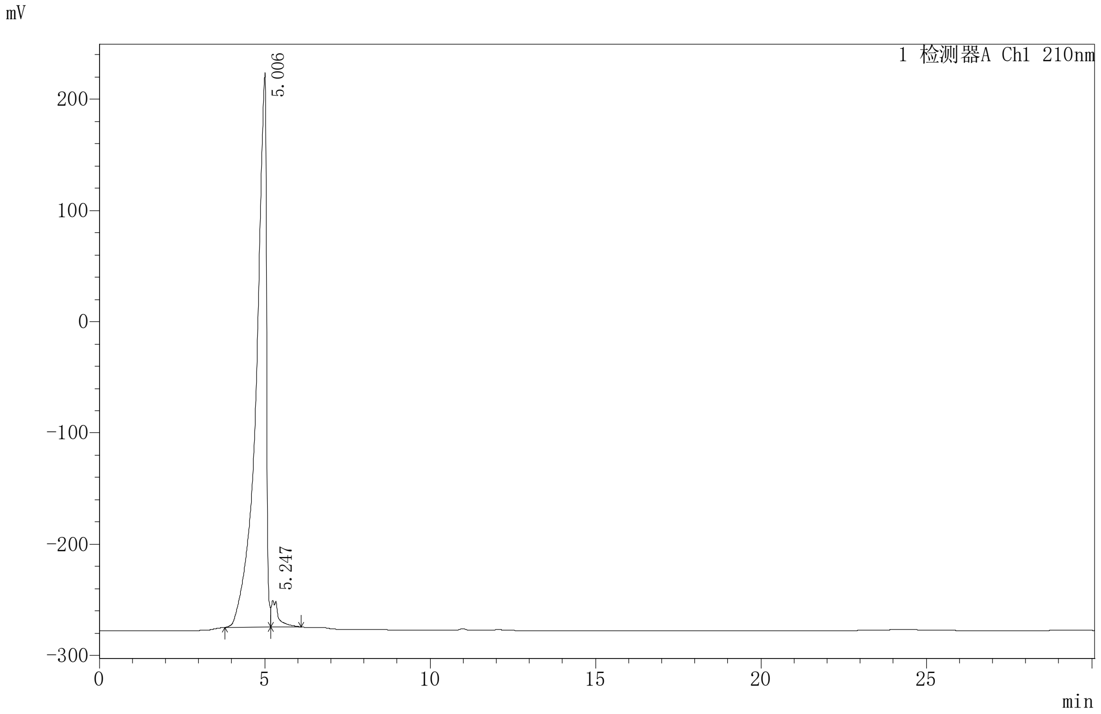

Figure S S1.10.(4) HPLC of compound 11

Table S1.4 HPLC of compound 11

Peak	Residual time(Min)	Area	Height	Concentration（%）	Area%
1	5.006	10573847	498694	96.644	96.644
2	5.247	367180	24197	3.356	3.356
		11058939		100.000	100.000

Methyl O-(methyl 2,3,4-tri-O-acetyl-β-D-glucopyranosyluronate)-(1→3)-(4,6-di-O-acetyl-2-acetamido-2-deoxy-β-D-glucopyranoside)-(1→4)-(methyl 2,3-di-O-acetyl-β-D-glucopyranosyluronate)-(1→3)-4,6-di-O-acetyl-2-acetamido-2-deoxy-D-glucopyranoside (12)

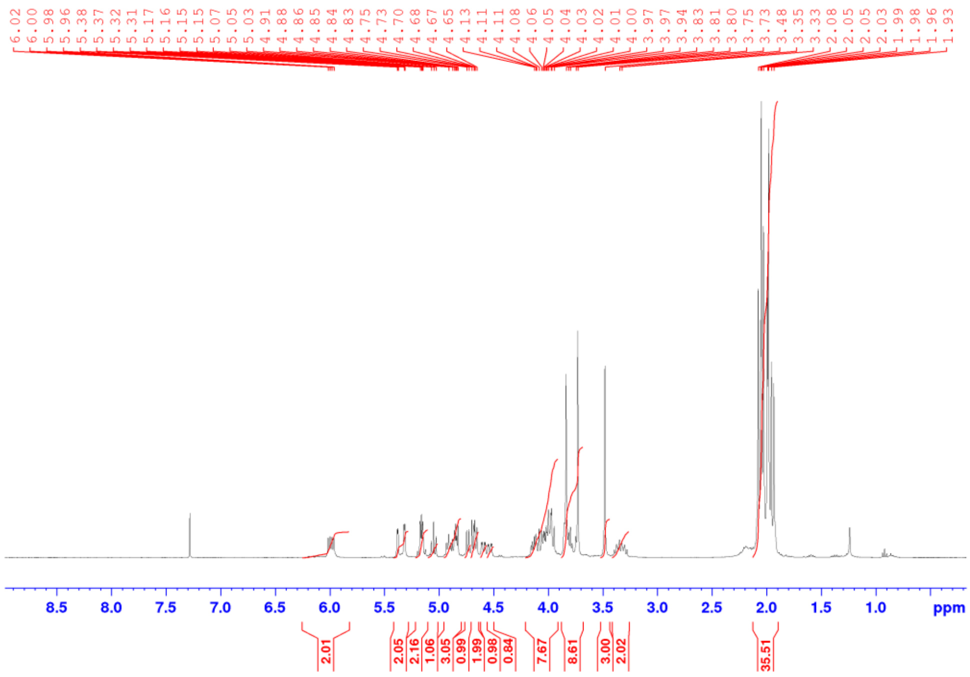

Figure S1.11.(1) ^{1}H NMR of the compound 12 (400MHz, $CDCl_3$, TMS)

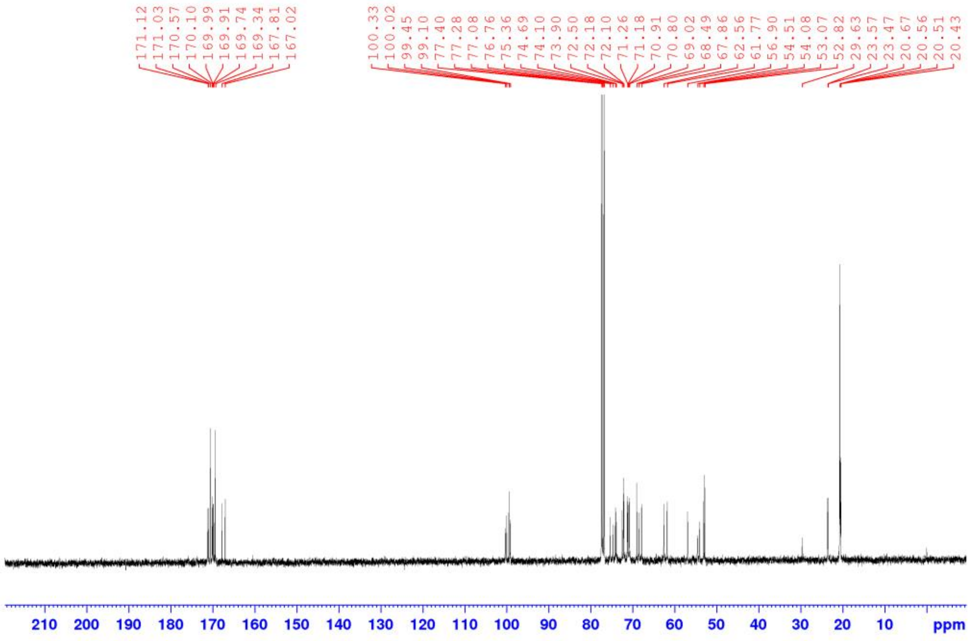

Figure S1.11.(2) ^{13}C NMR of the compound 12 (150MHz, $CDCl_3$, TMS)

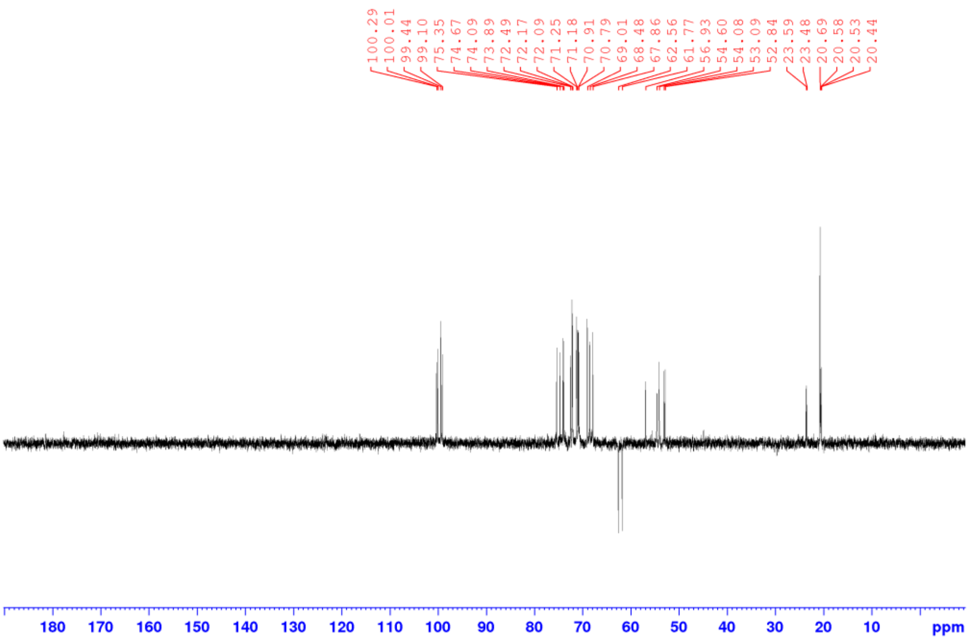

Figure S1.11.(3) DEPT-135 NMR of the compound 12 (150MHz, CDCl3, TMS)

Methyl O-(methyl 2,3,4-tri-O-acetyl-β-D-glucopyranosyluronate)-(1→3)-(4,6-di-O-acetyl-2-acetamido-2-deoxy-β-D-glucopyranoside)-(1→4)-(methyl 2,3-di-O-acetyl-β-D-glucopyranosyluronate)-(1→3)-(4,6-di-O-acetyl-2-acetamido-2-deoxy-D-glucopyranoside)-(1→4)-(methyl 2,3-di-O-acetyl-β-D-glucopyranosyluronate)-(1→3)-4,6-di-O-acetyl-2-acetamido-2-deoxy-D-glucopyranoside (13)

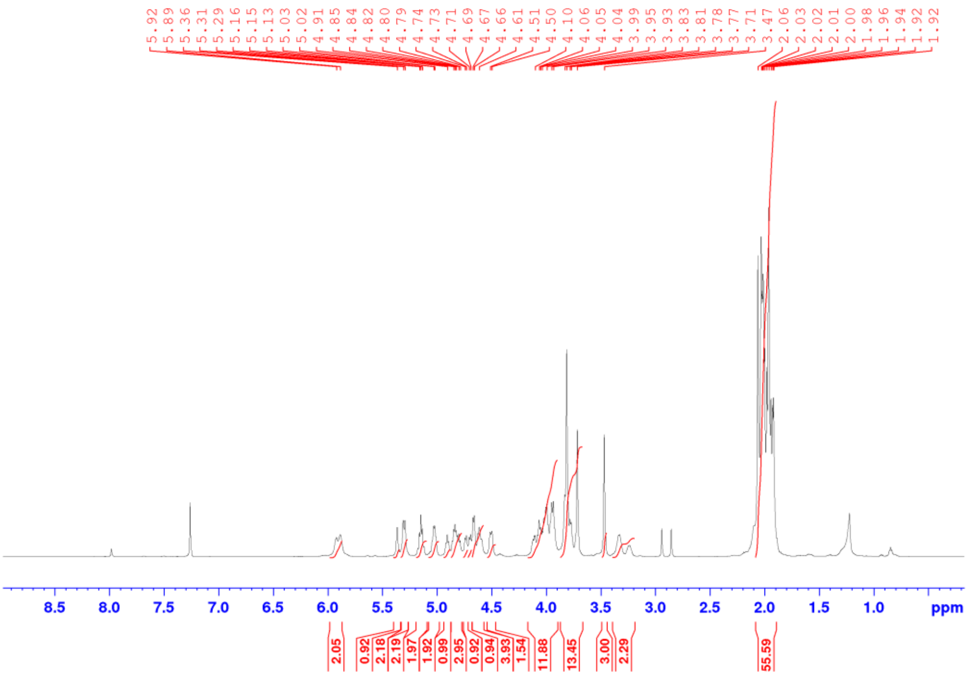

Figure S1.12.(1) ^{1}H NMR of the compound 13 (600MHz, $CDCl_3$, TMS)

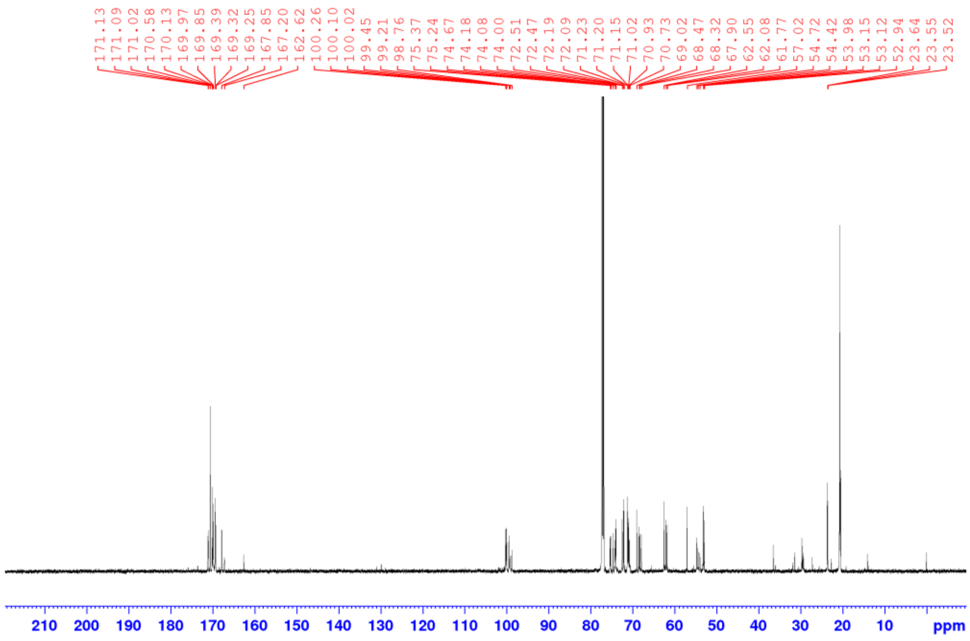

Figure S1.12.(2) ^{13}C NMR of the compound 13 (150MHz, $CDCl_3$, TMS)

Methyl O-(β-D-glucopyranosyluronate)-(1→3)-(2-acetamido-2-deoxy-β-D –glucopyranoside)-(1→4)- (β-D-glucopyranosyluronate)-(1→3)-2-acetamido-2-deoxy-β-D –glucopyranoside (14)

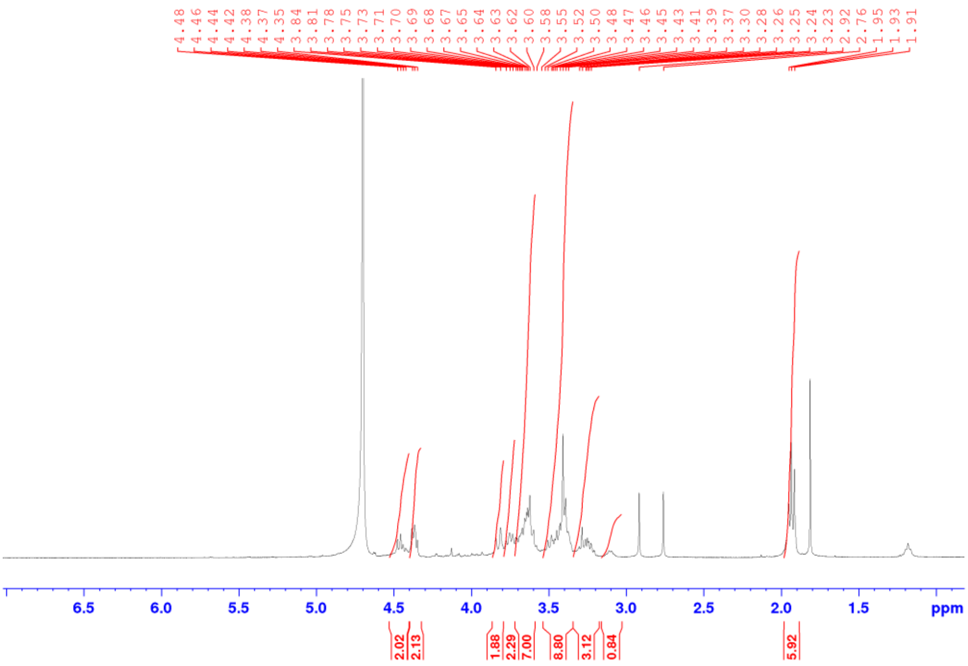

Figure S1.13.(1) ^{1}H NMR of the compound 14 (400MHz, D_2O, TMS)

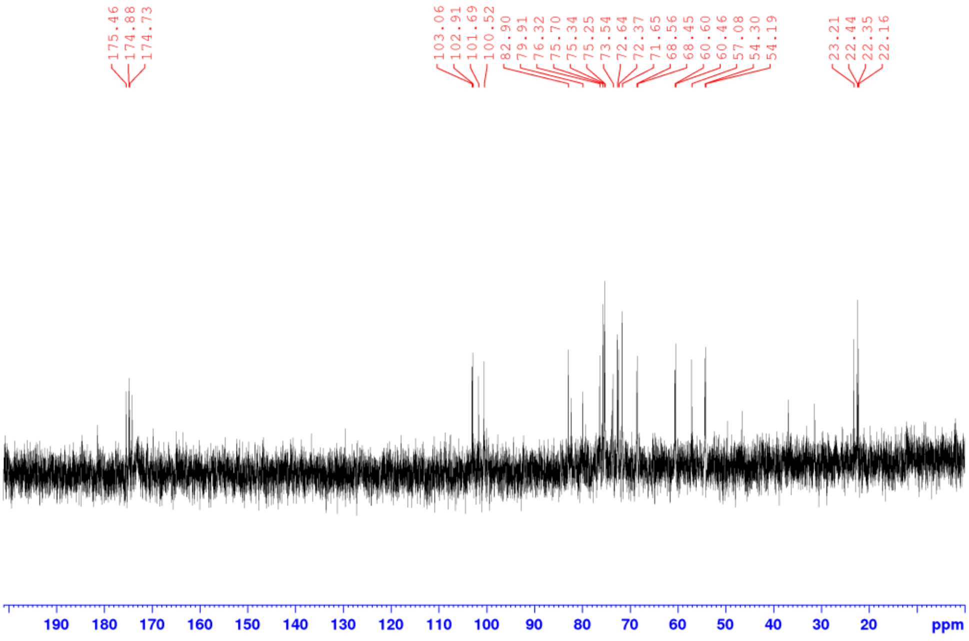

Figure S1.13.(2) ^{13}C NMR of the compound 14 (100MHz, D_2O, TMS)

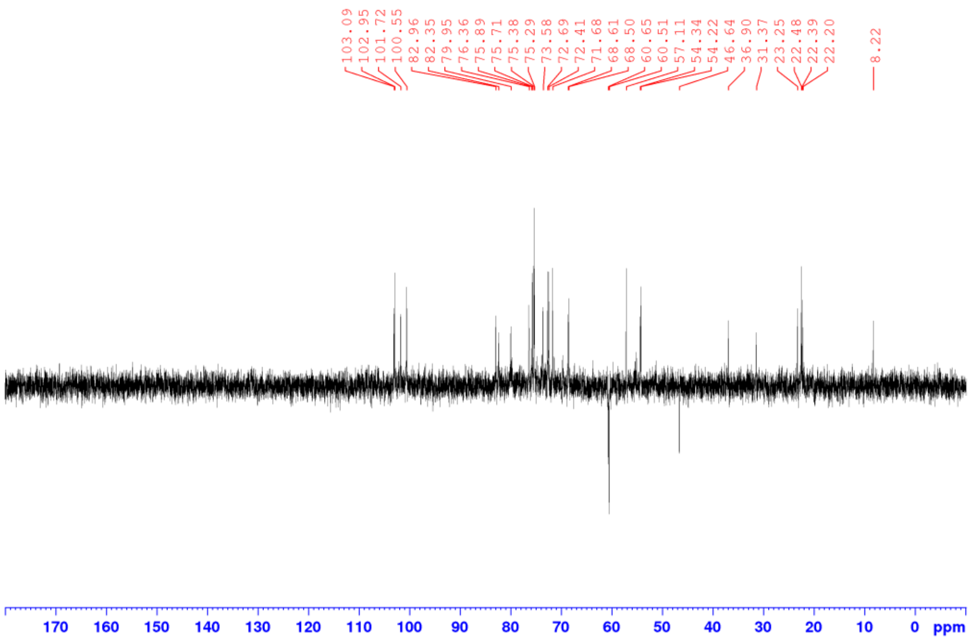

Figure S1.13.(3) DEPT-135 NMR of the compound 14(100MHz, D_2O, TMS)

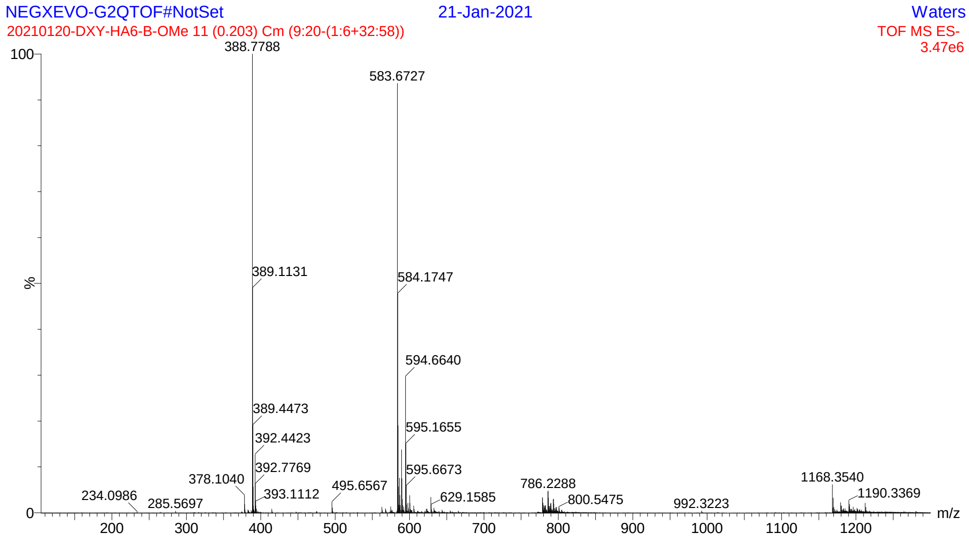

Figure S1.13.(4) HR-MS of the compound 14

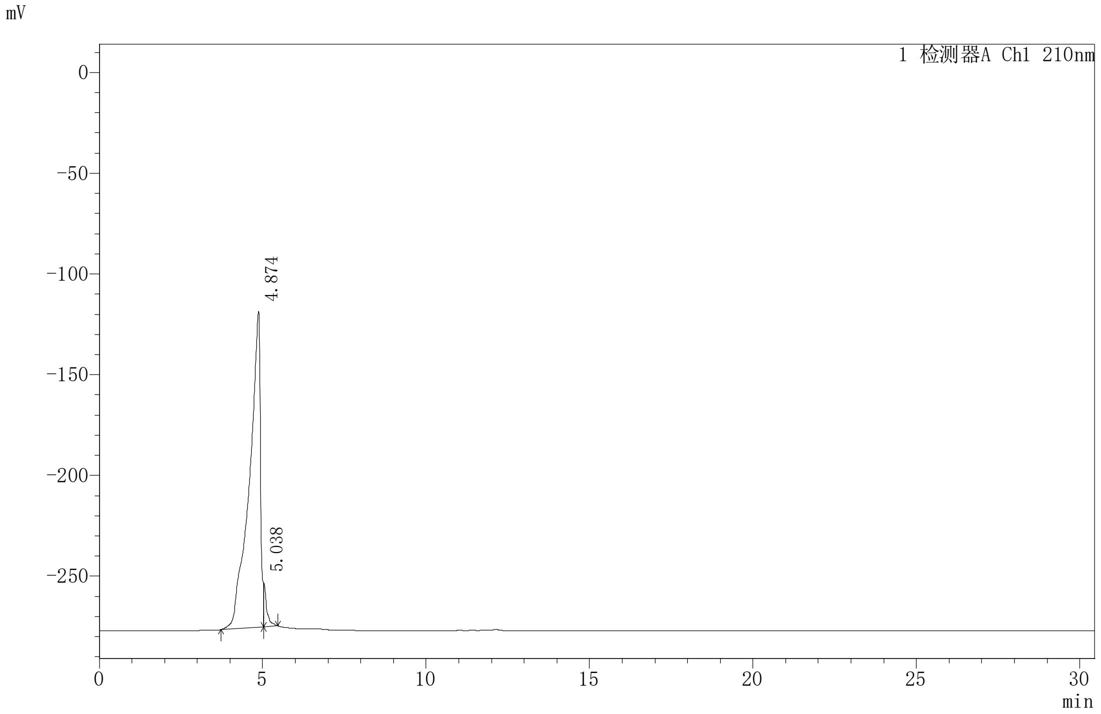

Figure S1.13.(5) HPLC of compound 14

Table S1.5 HPLC of compound 14

Peak	Residual time(Min)	Area	Height	Concentration（%）	Area%
1	4.874	3677179	156543	96.315	96.315
2	5.038	140674	21555	3.685	3.685
		3817853		100.000	100.000

Methyl O-(β-D-glucopyranosyluronate)-(1→3)-(2-acetamido-2-deoxy-β-D –glucopyranoside)-(1→4)- (β-D-glucopyranosyluronate)-(1→3)-(2-acetamido-2-deoxy-β-D –glucopyranoside)(1→4)- (β-D-glucopyranosyluronate)-(1→3)-(2-acetamido-2-deoxy-β-D –glucopyranoside) (15)

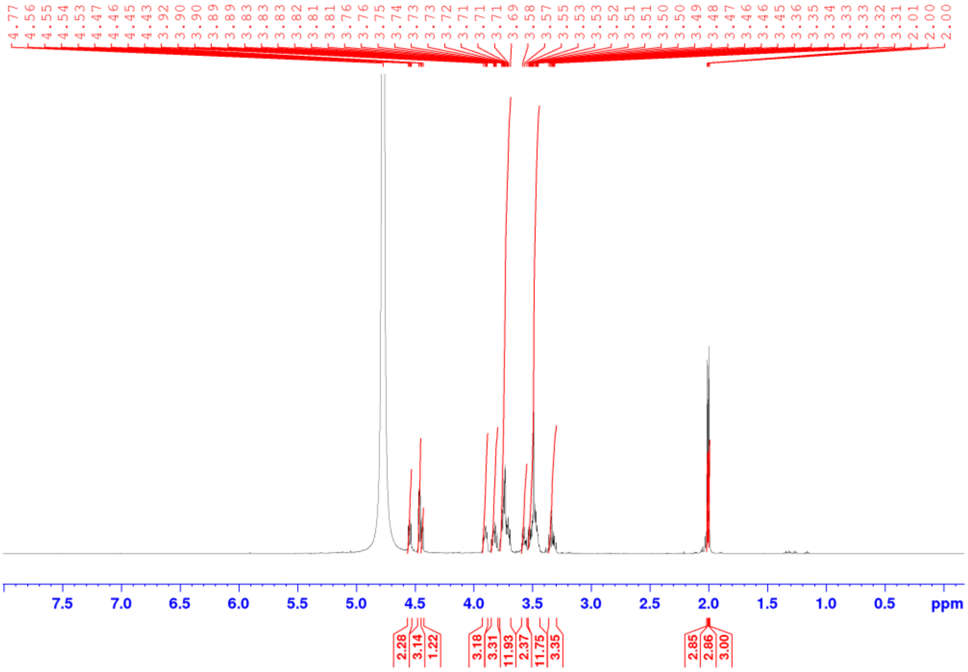

Figure S1.14.(1) ^{1}H NMR of the compound 15 (600MHz, D_2O, TMS)

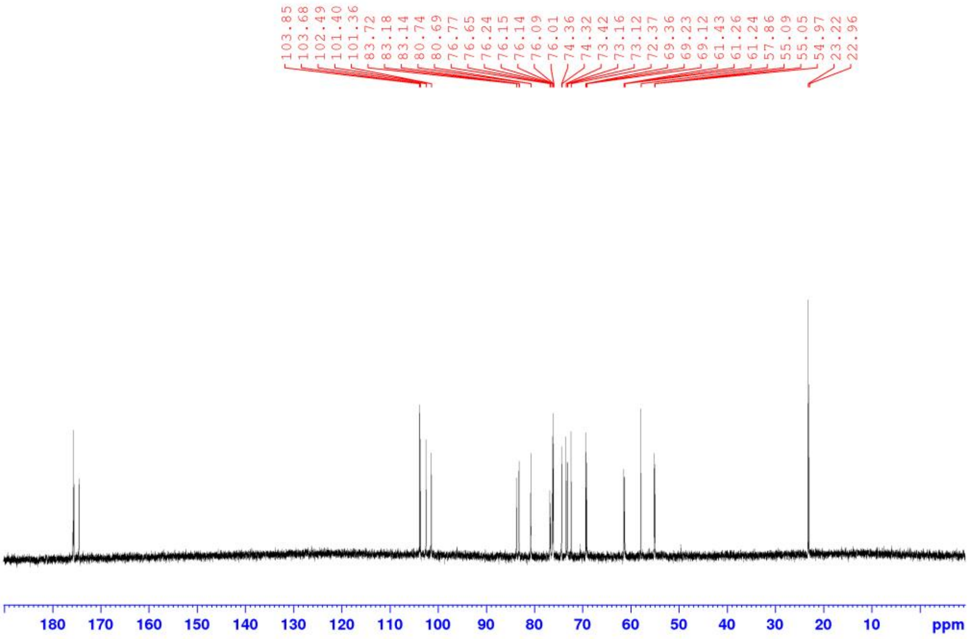

Figure S1.14.(2) ^{13}C NMR of the compound 15 (150MHz, D_2O, TMS)

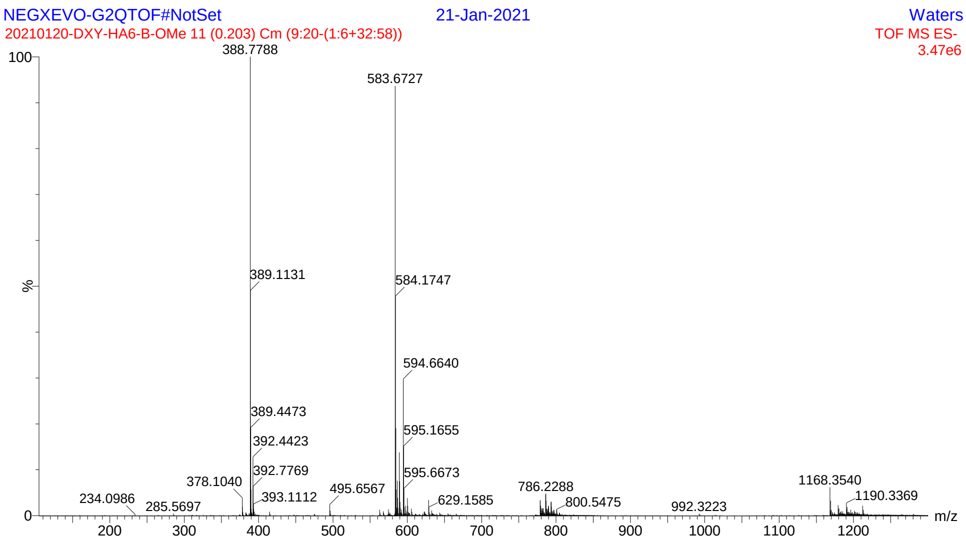

Figure S1.14.(3) HR-MS of the compound 15

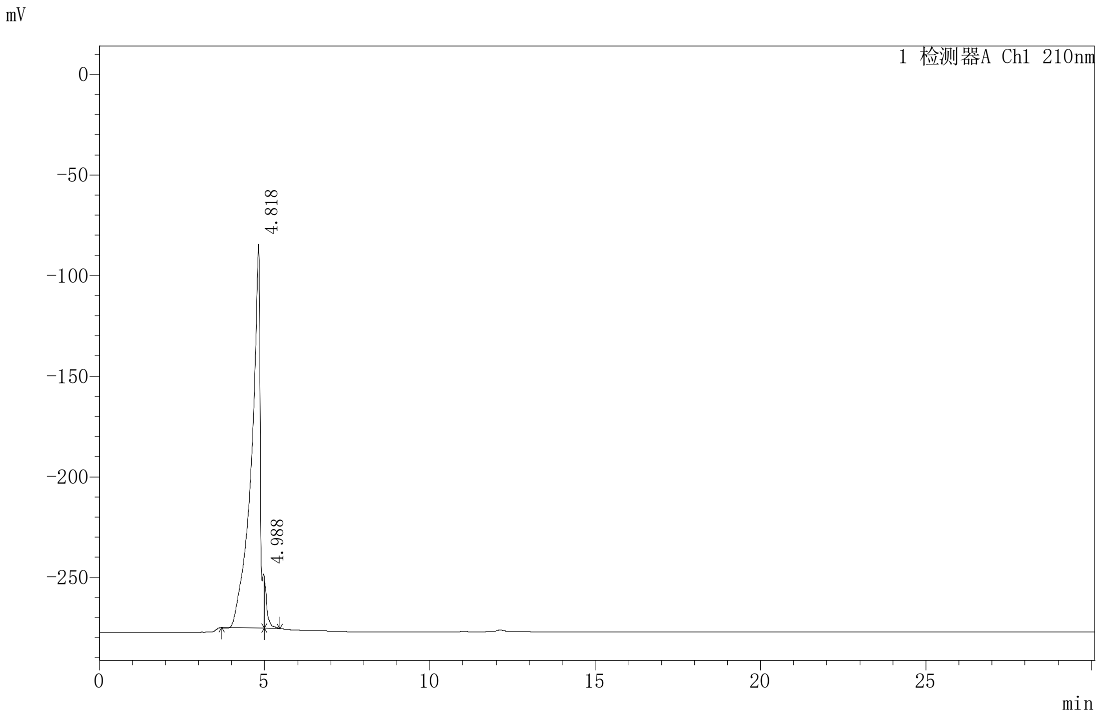

Figure S1.14.(5) HPLC of compound 15

Table S1.6 HPLC of compound 15

Peak	Residual time(Min)	Area	Height	Concentration（%）	Area%
1	4.818	3508696	190705	96.214	96.214
2	4.988	138078	25771	3.786	3.786
		3646774		100.000	100.000

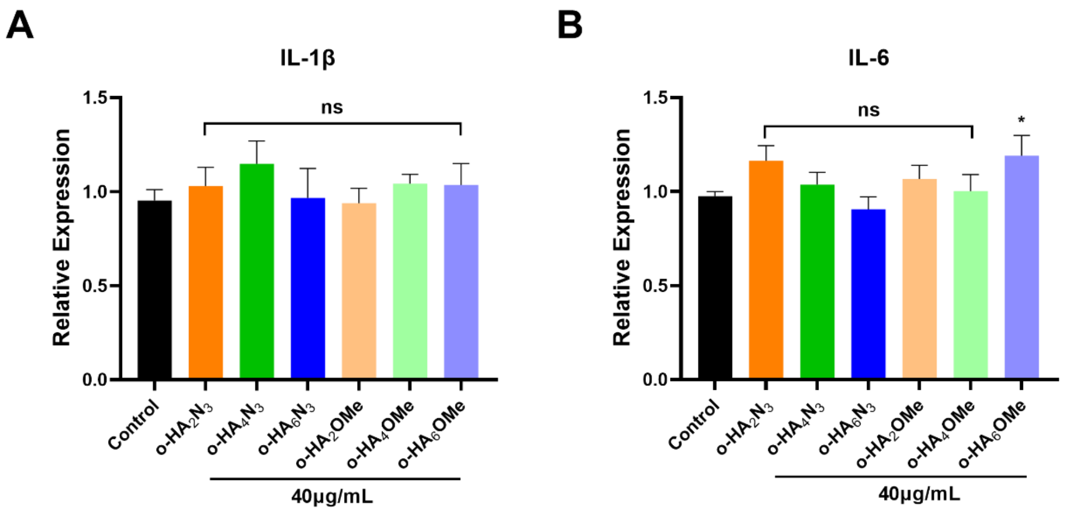

Figure S2 o-HA derivatives had no pro-inflammatory effect on THP-1 cells. RT-qPCR was used to confirm the mRNA expression levels of pro-inflammatory cytokines (IL-1β, IL-6) after co-culture with THP-1 cells of o-HAs derivatives(40μg/mL) for 24h. **(A, B)** RT-qPCR was used to confirm the mRNA expression levels of pro-inflammatory cytokines (IL-1β, IL-6). *p <0.05, *ns*=not statistically significant vs Control group.

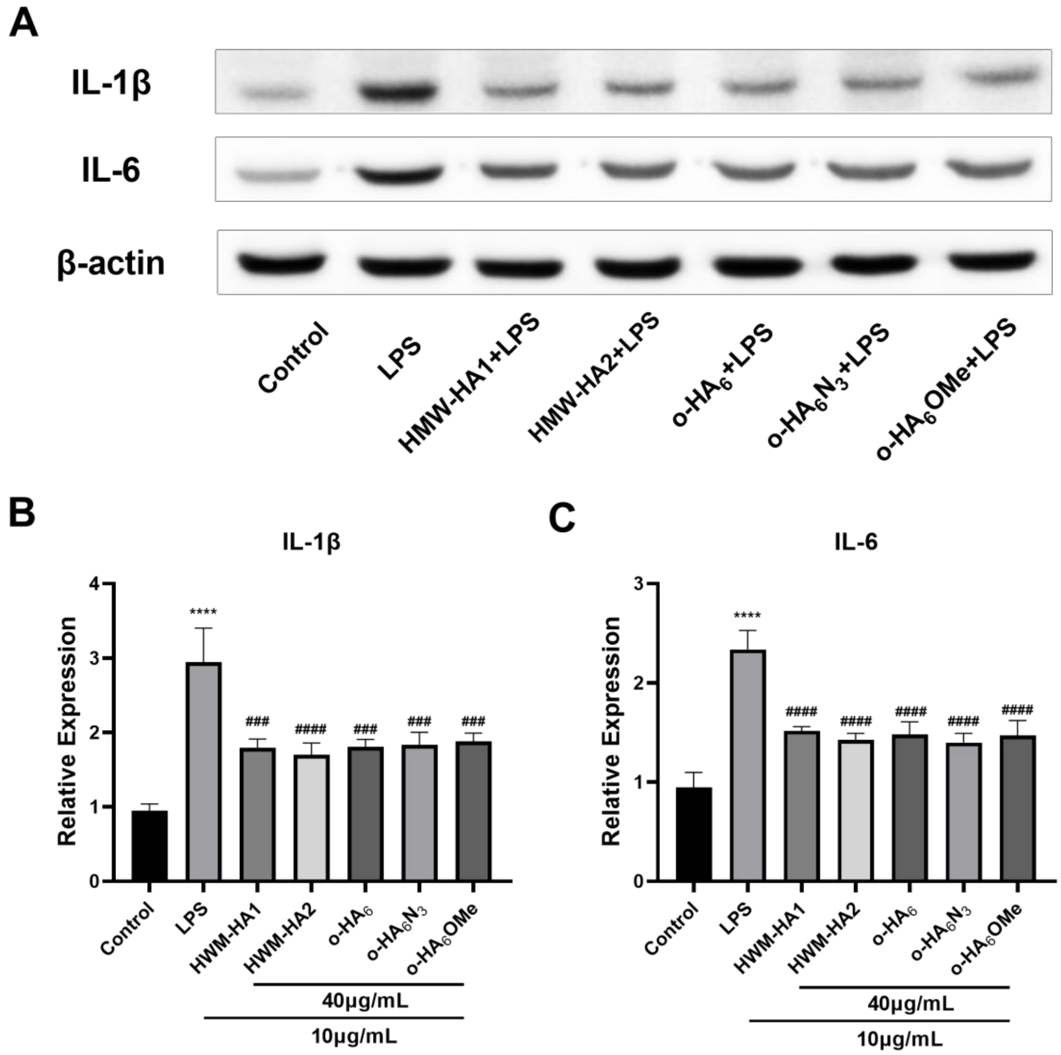

Figure S3 Different HA derivatives down-regulated IL-1β and IL-6 protein expression levels in LPS-induced inflammatory injury in ATDC5 cells. ATDC5 cells in the LPS group were stimulated with LPS (10 μg/mL) for 24 h and then cultured in the medium containing 10% FBS for another 24h. For different HA derivatives groups, ATDC5 cells were treated with LPS (10 μg/ml) for 24 h and HA derivatives (40 μg/mL) for an additional 24 h, respectively. **(A–C)** The protein levels of IL-1β and IL-6 were detected by Western blot. ****p <0.0001 vs Control group; $^{###}p$ <0.001, $^{####}p$ <0.0001 vs LPS group.

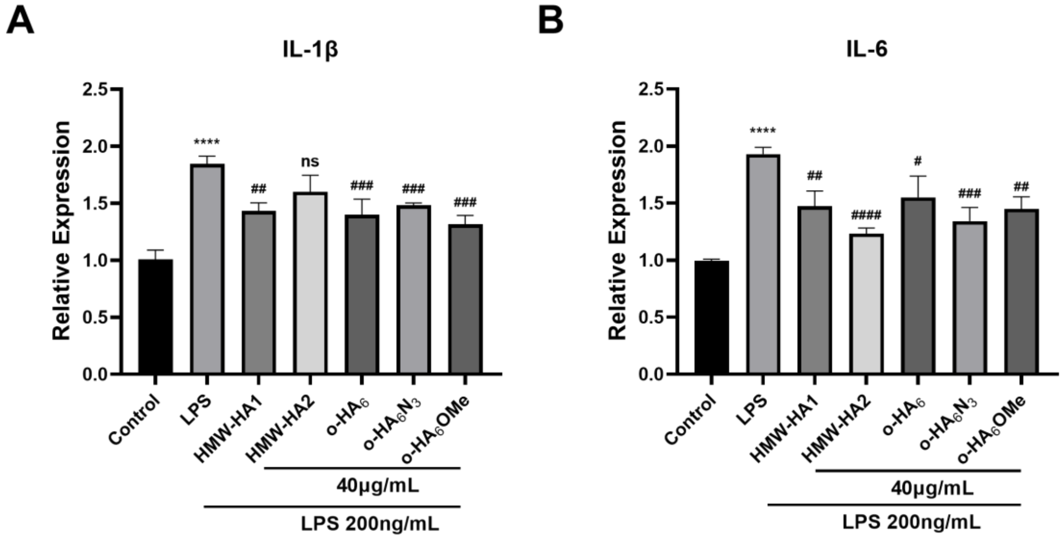

Figure S4 Different HA derivatives could alleviate the inflammatory injury of THP-1 cells induced by LPS. THP-1 cells in FBS concentration and CO_2 incubator conditions were the same as ATDC5 cells. PMA (25 ng/mL) induced THP-1 monocytes to differentiate into macrophages. After 48 h induction, THP-1 cells were incubated in RPMI-1640 containing LPS (final concentration 200ng/mL) or LPS plus different HA derivates (40μg/mL) for 24 h. **(A, B)** mRNA expression levels of IL-1β and IL-6 were detected by RT-qPCR. $^{****}p$ <0.0001 vs Control group; $^{\#}p$ <0.05, $^{\#\#}p$ <0.01, $^{\#\#\#}p$ <0.001, $^{\#\#\#\#}p$ <0.0001, ns=not statistically significant vs LPS group.

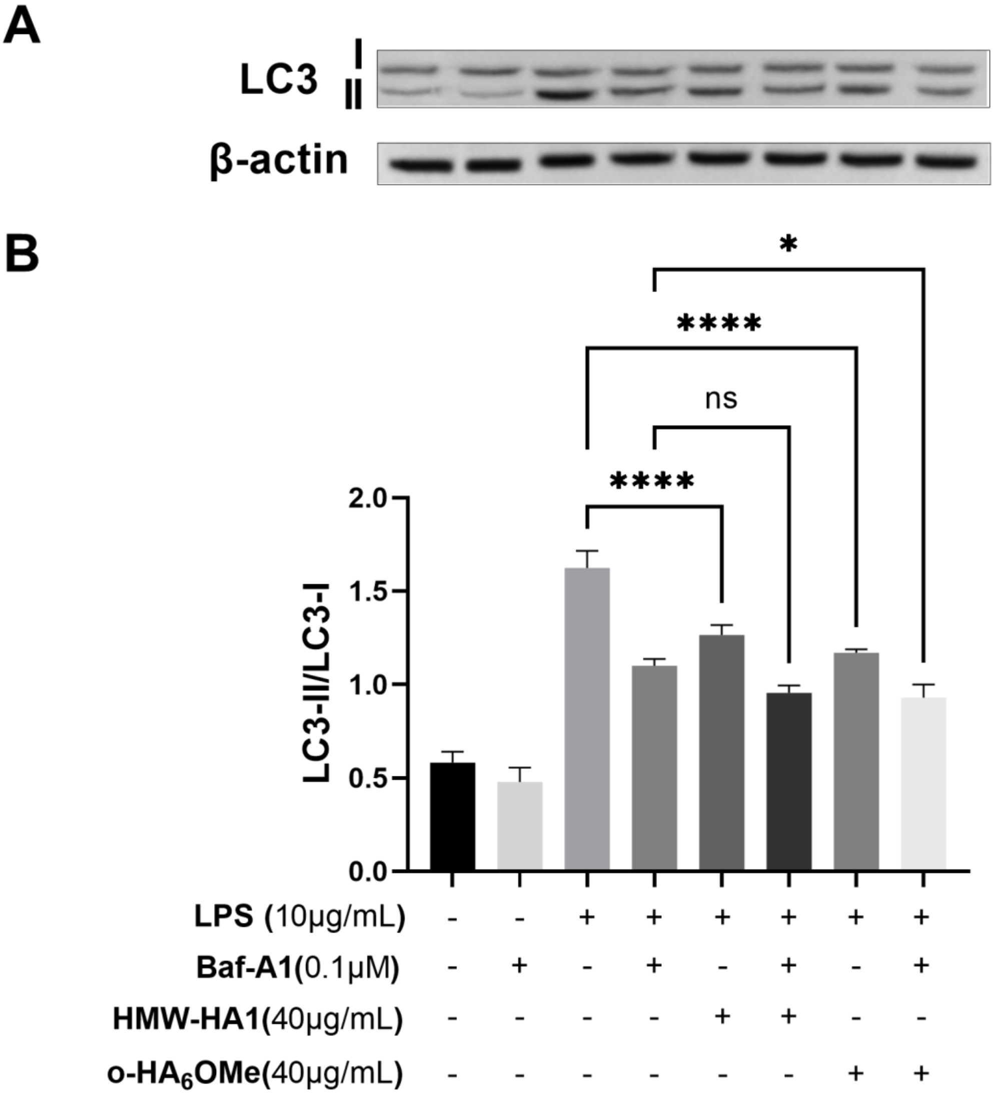

Figure S5 The ratio of LC3-II/LC3-I was determined by Western blot analysis after using autophagy inhibitor. ATDC5 cells in the LPS group were stimulated with LPS (10 μg/ml) for 24 h and then cultured in the medium containing 10% FBS for another 24h. For different HA derivatives groups, ATDC5 cells were treated with LPS (10 μg/ml) for 24 h and HA derivatives (40 μg/ml) for an additional 24 h, respectively. The medium always contained Baf-A1 at a final concentration of 0.1 μM during LPS injury and HA derivatives treatment. **(A, B)** LC3-II/LC3-I ratio were detected by Western blot analysis. ****p <0.0001, *p <0.05, ns=not statistically significant.
